# Mapping QTLs for water-use efficiency reveals the potential candidate genes involved in regulating the trait in apple under drought stress

**DOI:** 10.1186/s12870-018-1308-3

**Published:** 2018-06-26

**Authors:** Haibo Wang, Shuang Zhao, Ke Mao, Qinglong Dong, Bowen Liang, Chao Li, Zhiwei Wei, Mingjun Li, Fengwang Ma

**Affiliations:** State Key Laboratory of Crop Stress Biology for Arid Areas/Shaanxi Key Laboratory of Apple, College of Horticulture, Northwest A&F University, Yangling, 712100 Shaanxi China

**Keywords:** Apple, Quantitative trait loci (QTLs), Carbon isotope composition, Water-use efficiency (WUE), Candidate genes, Drought stress

## Abstract

**Background:**

Improvement of water-use efficiency (WUE) can effectively reduce production losses caused by drought stress. A better understanding of the genetic determination of WUE in crops under drought stress has great potential value for developing cultivars adapted to arid regions. To identify the genetic loci associated with WUE and reveal genes responsible for the trait in apple, we aim to map the quantitative trait loci (QTLs) for carbon isotope composition, the proxy for WUE, applying two contrasting irrigating regimes over the two-year experiment and search for the candidate genes encompassed in the mapped QTLs.

**Results:**

We constructed a high-density genetic linkage map with 10,172 markers of apple, using single nucleotide polymorphism (SNP) markers obtained through restriction site-associated DNA sequencing (RADseq) and a final segregating population of 350 seedlings from the cross of Honeycrisp and Qinguan. In total, 33 QTLs were identified for carbon isotope composition in apple under both well-watered and drought-stressed conditions. Three QTLs were stable over 2 years under drought stress on linkage groups LG8, LG15 and LG16, as validated by Kompetitive Allele-Specific PCR (KASP) assays. In those validated QTLs, 258 genes were screened according to their Gene Ontology functional annotations. Among them, 28 genes were identified, which exhibited significant responses to drought stress in ‘Honeycrisp’ and/or ‘Qinguan’. These genes are involved in signaling, photosynthesis, response to stresses, carbohydrate metabolism, protein metabolism and modification, hormone metabolism and transport, transport, respiration, transcriptional regulation, and development regulation. They, especially those for photoprotection and relevant signal transduction, are potential candidate genes connected with WUE regulation in drought-stressed apple.

**Conclusions:**

We detected three stable QTLs for carbon isotope composition in apple under drought stress over 2 years, and validated them by KASP assay. Twenty-eight candidate genes encompassed in these QTLs were identified. These stable genetic loci and series of genes provided here serve as a foundation for further studies on marker-assisted selection of high WUE and regulatory mechanism of WUE in apple exposed to drought conditions, respectively.

**Electronic supplementary material:**

The online version of this article (10.1186/s12870-018-1308-3) contains supplementary material, which is available to authorized users.

## Background

Agricultural crops are facing severe water shortages in many parts of the world [1]. This includes the Loess Plateau of China, where a unique solar resource and temperate climate have helped that region become one of the most productive places in which to grow apple (*Malus* × *domestica*). However, limited water availability threatens the sustainable production of apple in that region [2]. For such arid and semi-arid locations, improving water-use efficiency (WUE) can be an effective approach reducing production losses caused by drought stress [3]. The preferred method would be to develop crop varieties with high WUE [4].

Many studies illustrate the complexity of WUE. Stomatal activity has a key role in regulating WUE because it can control evaporation rates and CO_2_ uptake [5]. Stomatal movement in response to drought could be determined by various factors, e.g., abscisic acid (ABA) and Ca^2+^ [6]. Adjusting stomatal density is another adaptive response to water deficits [7]. In apple, maintaining high WUE under drought may be achieved by supporting normal functioning of the photosynthesis system, reducing the production of reactive oxygen species (ROS), and enhancing the net photosynthesis rate, in addition to driving stress-signaling and drought-responsive proteins [8]. Several regulatory genes have already been identified. Among these, *ERECTA* in *Arabidopsis* modulates transpiration efficiency by modifying stomatal density, epidermal cell expansion, mesophyll cell proliferation, and cell–cell contacts within the leaf [9]. Overexpression of *Arabidopsis HARDY* in rice can enhance photosynthesis and reduce transpiration, which then leads to improved WUE and drought tolerance [10]. *GTL1* in *Arabidopsis* represses *SDD1* to regulate stomatal density and, ultimately, WUE [7]. In *Arabidopsis*, an amino acid substitution caused by a single nucleotide change in *MPK12* leads to a reduction in ABA-inhibition of stomatal opening and WUE, but an increase in guard cell size and short-term sensitivity to higher vapor pressure deficit (VPD) [11]. Overexpression of *PdEPF1* enhances poplar WUE and water deficit tolerance [12]. Other examples include *AGO*s in apple [13].

High-density genetic linkage maps built using single nucleotide polymorphisms (SNPs) are a key tool toward the implementation of marker-assisted selection (MAS) of favorable alleles or traits in breeding [14]. Several studies have focused on developing potential markers or loci linked with desired traits with the help of SNP-based genetic maps [15–17]. A benefit of such high-density maps is that they can narrow the confidence intervals of QTLs for target traits, thus increasing the efficiency to find candidate genes. For example, based on dense SNP genetic maps of apple, potential genes for fruit texture, acidity and shape have been revealed, respectively [18–20]. Although researchers have identified QTLs for the indices of photosynthetic gas exchange under atmospheric drought stress, as well as the production of vegetative tissue and fruit under soil water-deficit conditions, the associated genes have not been screened because the density of single sequence repeats (SSRs) on their genetic maps has been insufficient [21, 22].

Carbon isotopic composition (δ^13^C) is an indicator that integrally reflects the endogenetic plant physiological and exogenetic environmental properties related to long-term carbon fixation by photosynthesis [23]. Since a significant correlation was first reported between δ^13^C and WUE in several genotypes of *Triticum aestivum* [23], this has subsequently been verified for *Sinapis alba*, *Brassica napus*, *B. campestris*, *Pisum sativum*, *Tritieum durum*, *T. aestivum* [24], *Arabidopsis* [25], *Quercus robur* [26], as well as for 10 *Malus* rootstocks [27] and 31 apple cultivars [28]. Glenn reported that leaf carbon isotopic discrimination (Δ^13^C), a negative linear correlation with δ^13^C, had a significantly negative correlation with apple WUE, and could be used as a high throughput and cost-efficient method to assess WUE in apple [29]. Mapping QTLs for Δ^13^C also has facilitated the identification of *ERECTA*, a key regulator of transpiration efficiency in *Arabidopsis* [9]. All of these reports indicate that WUE is significantly and positively correlated with δ^13^C, and suggest that this is suitable parameter for evaluating whole-plant WUE, especially of numerous individuals of a population.

For the research described here, we constructed an apple hybrid population of ‘Honeycrisp’ × ‘Qinguan’. ‘Honeycrisp’ is from the cross of ‘Keepsake’ and an uncertain variety [30]. And ‘Qinguan’ is the offspring of ‘Golden Delicious’ × ‘Jiguan’. ‘Honeycrisp’ and ‘Qinguan’ show obvious difference in WUE [28], and their segregating population provides good material for exploring apple WUE via quantitative genetics. To improve our understanding of the genetic determination of WUE in apple under drought stress, we constructed a high-density genetic map using this F_1_ population, and then performed QTL mapping of δ^13^C. SNPs for map construction were developed by Restriction site-Associated DNA sequencing (RADseq), which has been widely used for barley [31], eggplant [32], pear [33], grape [34] and apple [19]. Based on the QTLs for δ^13^C as shown on our new genetic map, we were able to identify potential markers and genes involved in WUE regulation in apple under drought stress. Our finding will be helpful for future MAS and genetic improvements of WUE in apple, a crop that is increasingly threatened by water deficit.

## Methods

### Plant material and watering treatments

The mapping population was F_1_ individuals from the cross of ‘Honeycrisp’ (HC) × ‘Qinguan’ (QG), which was performed in 2007 and produced 1048 seedlings. They were then individually grafted onto M_26_ rootstock in 2009 and planted in the hybrid nursery of Northwest A&F University, Yangling (34°20’N, 108°24’E), Shaanxi Province, China.

We evaluated δ^13^C of 182 individuals from the mapping population in 2014, 145 individuals in 2015, and their parent cultivars. The 145 individuals evaluated in 2015 were in common with those used in 2014. For each test year, we obtained 14 replicate trees of each hybrid and 30 replicates of their parents by bud-grafting onto *Malus hupehensis* (Pamp.) Rehd. rootstock in March. All were planted in plastic pots (38 × 23 cm) filled with a local 5:1 (v:v) loess soil:sand medium. The seedlings were then put into a greenhouse under natural light, with a temperature range of 20 to 35 °C and relative humidity 55 to 75%. The 60-d watering treatments began when the young trees were approximately 50 cm tall. The two contrasting irrigation regimes were 1) well-watered (WW), in which seven plants of each hybrid and 15 plants of each parent were watered to maintain 65 to 75% field capacity; and 2) drought-stressed (DS), in which the field capacity for seven plants of each hybrid and 15 plants of each parent was maintained at 45 to 55% by watering [28].

### DNA extraction, RAD libraries construction and sequencing

Genomic DNA in young leaves sampled from 362 individuals and their parents (gDNA) was isolated using a DNAsecure Plant Kit (Tiangen Biotech Co., Beijing, China). The DNA quantity and quality were determined with a NanoDrop 2000 spectrophotometer (Thermo Fisher Scientific, Wilmington, NC, USA) and by electrophoresis in 1.0% agarose gels with a λDNA/HindIII marker.

The RAD libraries for marker development were constructed as previously described [31, 33]. Briefly, this procedure followed nine main steps: digesting gDNA with restriction enzyme *Eco*RI (New England BioLabs, or NEB, Ipswich, MA, USA); adding P1 adapters (Illumina, San Diego, CA, USA) to each digested fragment by T4 DNA ligase (NEB); pooling and shearing these fragments; purifying sheared DNA fragments using QIAquick PCR Purification kit; selecting 200- to 400-bp DNA fragments by agarose gel electrophoresis; repairing and flattening fragment ends using a Quick Blunting kit (NEB); adding the overhang (A 3′-dA) to these fragments by a dA-tailing module (NEB); adding P2 adapters (Illumina) to these fragments; and enriching tagged DNA by PCR-amplification. Sequencing was performed on an Illumina Hiseq2500 platform at Novogene Co. (Beijing, China), applying PE250 strategy according to the Illumina protocol.

### RADseq-based SNP marker development and map construction

To ensure the high quality of further analyses, we adjusted the raw sequencing data as follows. After eliminating reads with adapter sequences, we removed any reads containing unidentified bases that comprised > 10% of their sequences. We also removed reads with > 50% low quality bases (quality value ≤5). The Q20 and Q30 values (correct base-recognition rates of 99 and 99.9%, respectively) were employed to evaluate the quality of these clean data. The paired-end reads in clean data of parents and their hybrids was aligned with Apple Genome v1.0 [35] using BWA software [36]. The formats of the alignment results were converted with SAMtools [37] to SAM/BAM files. After the orders were sorted and repetitions deleted, we selected reads aligned at unique positions on the reference genome. The SAMtools were also used to detect SNPs from filtered BAM files. Reducing the occurrence of false-positive SNPs caused by erroneous sequencing required that the base supporting number be at least 10 for parents, and more than 5 for the hybrids. This could also be accomplished by aligning in repeat regions, in which the base supporting number should be no more than 5000 for both parents and hybrids. After, the heterozygous and homozygous SNPs were counted, and their rates were determined. By eliminating monomorphic markers according to the genotypes of the parents, three segregating types of markers: lm × ll, nn × np and hk × hk, were acquired.

Genetic linkage maps were constructed with JoinMap 4.1 [38]. The regression mapping algorithm and Kosambi’s mapping function to calculate genetic distances were used as basic sets. A logarithm of the odds (LOD) score of 6.0 was utilized to divide linkage groups (LGs), and markers were filtered with designated missing values (20%). Those markers with distorted segregation (*p* < 0.01 in Chi-square test) were discarded and any seedling in mapping population were excluded if they exhibited several double-recombination events. LGs were drawn using MapChart 2.3 [39], and were named according to ‘HC no.’, ‘QG no.’ and ‘LG no.’ (HC, ‘Honeycrisp’; QG, ‘Qinguan’; and LG, the integrated HC × QG map).

### Phenotyping and QTLs mapping

The ninth leaf from the shoot apex was sampled from each selected tree at the end of the watering experiment [27, 28]. For each treatment type, five leaves from five plants of each genotype were pooled as one sample. They were oven-dried first at 105 °C for 0.5 h, then at 70 °C for approximately 72 h to a constant weight before being ground and filtered through a sieve (80 holes per cm^2^). The δ^13^C for each sample was determined with an elementary analysis-isotope ratio mass spectrometer (Flash EA 1112 HT-Delta V Advantages, Thermo Fisher Scientific) and was calculated as δ^13^C(‰) = [(R_VPDB_/R_sample_)-1]*1000, where R_VPDB_ and R_sample_ were the ^13^C/^12^C values for the international standard VPDB (Vienna Peedee Belemnite) and sample, respectively.

The QTL analysis was conducted with MapQTL 6.0 [40], and was initially run with interval mapping (IM) computation. The significant LOD threshold of QTLs was determined through calculations using 1000 permutations. Any QTLs with LOD scores at the 95% genome-wide threshold were significant [40]. Multiple QTL model (MQM) mapping was then performed using loci nearest the QTL peaks as co-factors. Designations for QTLs were based on trait (δ13C), treatment (WW or DS), year (‘14’ or ‘15’), and genetic position (i.e., number of linkage group). For LGs that had more than one QTL, the designation also included a dot and number suffix.

### Assay-based SNP marker genotyping using Kompetitive allele-specific PCR (KASP)

Based on stable QTLs for δ^13^C under drought stress condition between years, three stable SNP markers close to their LOD score peaks were selected. Using 50-bp flanking sequences of these SNPs in Apple Genome v1.0, we designed and synthesized KASP primers (Additional file 1: Table S1) before InnovaChip™ micro-fluidic chips were made by CapitalBio Co. (Beijing, China). The final reaction system of KASP (1.0 μL) contained 20 ng of template DNA and 0.5 μL of KASP v4.0 2X Master Mix (LGC, Hoddesdon, UK). Reaction solutions for the different genotypes were loaded onto the micro-fluidic chip, which was then centrifuged at 3000 rpm for 1 min. The KASP was completed using an FP4 PCR instrument (CapitalBioTech, Beijing, China). The PCR programs included one cycle of 95 °C for 15 min; 10 cycles of 95 °C for 20 s and 61 °C for 60 s, with the annealing temperature reduced by 0.6 °C per cycle; followed by 26 cycles of 95 °C for 20 s and 55 °C for 60 s. Fluorescence signals were detected with a microarray chip scanner (CapitalBioTech) after the temperature of the reaction products was reduced to 37 °C.

The δ^13^C phenotypic values of seven commercial apple cultivars exposed to drought stress [28] were used here to compare among phenotypes and genotypes identified by KASP.

### Screening of candidate genes

The corresponding intervals for targeted QTLs were mapped on Apple Genome v1.0, according to the associated markers. Genes in those regions were downloaded from GDR (Genome Database for Rosaceae) website (http://www.rosaceae.org/species/malus/malus_x_domestica/genome_v1.0). Their chromosomal locations and functional annotations were obtained from the Plaza 3.0 website (http://bioinformatics.psb.ugent.be/plaza). Gene Ontology (GO) annotation results of candidate genes were plotted on WEGO website (http://wego.genomics.org.cn/).

### Expression analysis of orthologs in *Arabidopsis* of candidate genes under drought stress

The file for the *Arabidopsis* proteome (TAIR10_pep_20101214) was obtained from the TAIR database (http://www.arabidopsis.org). Using the putative protein sequence of each candidate gene as a query, we performed a local blast (blastp method) to search the homologous genes for corresponding proteins in *Arabidopsis* with software BioEdit v7.0.9.0. The transcriptome data (Affymetrix microarray data; [41]) for drought-stressed plants of *Arabidopsis* (GSE5624) was acquired from the National Center for Biotechnology Information database (http://www.ncbi.nlm.nih.gov). After making one-one correspondences of gene IDs and log2-transformation of expression data, we drew hierarchical clustering heatmaps with MeV v4.9 to present the expression patterns of these orthologs under drought conditions. Differentially expressed genes that demonstrated at least a two-fold change (|log base 2 of fold-change| > 1) of average expression level of each gene by comparing the sample taken at 0.25 h with those examined from six other time points.

### Association network predictions for candidate genes

All of the protein sequences for candidates were submitted to STRING v10.0 (http://string-db.org). After finishing the blast step with “*Arabidopsis thaliana*” specified, those genes with the highest scores (Bitscore) were retained to construct the interacting network.

### RNA extraction and real-time quantitative RT-PCR (qRT-PCR) analysis

To analyze the change in expression for selected genes, we sampled the ninth leaves from the shoot apices of ‘Honeycrisp’ and ‘Qinguan’ plants after they were exposed to the two irrigation regimes. Total RNA was extracted with an RNAprep Pure Plant Kit (Tiangen). The purity and concentration of these extracted RNAs were evaluated by gel electrophoresis and a NanoDrop 2000 spectrophotometer, respectively. The cDNA of each sample was obtained through reverse-transcript of 1 μg of total RNA with a PrimeScript RT Reagent Kit (TaKaRa, Dalian, Liaoning, China). All qRT-PCR assays were performed in 20-μL reaction mixtures with 10 μL of SYBR® Premix Ex Taq™ (TaKaRa) using a BIO-RAD iQ5 instrument (Hercules, CA, USA) [42]. Samples were pooled from five plants per genotype for each treatment to make one biological replicate. Three replicates tested for each sample. The △Ct values were calculated by using apple *EF-1α* as our endogenous control according to Wang et al. (2011) [43]. All primers for qRT-PCR are detailed in Additional file 1: Table S1. The differences of gene expression levels between well-watered and drought-stressed plants for each cultivar were analyzed by independent-sample t-tests via SPSS v.16.0 (SPSS, Inc. Chicago, IL, USA). A *p*-value < 0.05 indicated a significant difference, and data were presented as the mean ± standard deviation of three replicates.

## Results

### RAD sequencing and SNP discovery

A total of 400.61 Gb clean bases with high-quality (Q20 ≥ 92.55% and Q30 ≥ 85.00%) and 1335.06 M clean reads were obtained. These data included 23.31 Gb from HC female parent (76.95 M reads), 20.15 Gb were from QG male parent (66.93 M reads), and total 357.15 Gb from progeny (1191.18 M reads). For individual of progeny, the maximum and minimum clean bases were 3.02 and 0.52 Gb, with 10.07 and 1.75 M reads, respectively. By comparing the Apple Genome v1.0, total clean bases aligned to apple reference genome accounted for genome size ranging from 0.71 X to 4.07 X for individuals of progeny, and 31.41 X (HC) and 27.16 X (QG) for parents. The sequencing depth of RAD tags varied from 8.76 X to 23.88 X for each individual. For parents, the depths were 77.75 X (HC) and 72.24 X (QG), respectively (Table 1).

**Table 1:** Quality evaluation of RAD-seq data for parents and progeny

Sample		Clean bases (Gb)	Clean reads (M)	Q20 (%)	Q30 (%)	GC content (%)	Coverage of genome^a^(X)	Depth of RAD tags^b^ (X)
Parents	Honeycrisp	23.31	76.95	95.38	89.75	37.42	31.41	77.75
	Qinguan	20.15	66.93	93.73	86.91	38.47	27.16	72.24
Progeny	Average	1.05	3.50	94.08	87.30	37.64		
	Maximum	3.02	10.07	96.53	91.75	40.14	4.07	23.88
	Minimum	0.52	1.75	92.55	85.00	35.78	0.71	8.76
	Total	357.15	1191.18					
All samples		400.61	1335.06					

^a^ Coverage of genome meant the proportion of total clean bases aligned to apple reference genome accounted for the genome size. The size of apple reference genome was 742.3 Mb estimated by Velasco et al.[35]. ^b^Depth of RAD tags meant the sequencing depth of filtered RAD tags

After deleting false-positive SNPs caused by erroneous sequencing or aligning in repeat regions, we obtained numerous high-fidelity SNPs (Table 2). For these SNPs, heterozygous loci in itself and homozygous loci but polymorphic amongst individuals of progeny were determined. The results showed that 533,016 (72.75%) loci in HC, 560,466 (73.97%) in QG and average 222,337 (74.89%) in individuals of progeny were homozygous. In contrast, fewer heterozygous loci were detected in HC (199,669, 27.25%), QG (197,270, 26.03%) and their progeny (average 74,564, 25.11%). Thus, based on the SNPs detected in the parents, we could identify the 349,085 polymorphism markers.

**Table 2:** Statistics of SNP discovered by RAD tag sequencing of parents and their progeny

Sample		Number of detected SNPs	Number of homozygous SNPs	Homozygous rate (%)	Number of heterozygous SNPs	Heterozygous rate (%)
Honeycrisp		732,685	533,016	72.75	199,669	27.25
Qinguan		757,736	560,466	73.97	197,270	26.03
Progeny	Average	296,901	222,337	74.89	74,564	25.11
	Maximum	609,057	450,471		165,776	
	Minimum	98,209	75,132		23,077	
	Total	100,946,295	75,594,513		25,351,782	
All samples		102,436,716	76,687,995		25,748,721	

### Map construction

Various thresholds were used to check abnormal bases, filter markers with designated missing values (20%), and discard any markers with distorted segregation (*p* < 0.001 in Chi-square test). Twelve seedlings of mapping population were excluded because they exhibited several double-recombination events. Using a final mapping population of 350 seedlings, we constructed an integrated map consisting of 10,172 markers and spanning 2430.52 cM. This included 4421 lm × ll-type markers, 4688 nn × np-type markers, and 1603 hk × hk-type markers. Among these, 5351 (4421 lm × ll plus 930 hk × hk) constituted linkage groups on the HC map with a total genetic distance of 1837.61 cM and 0.34 cM per marker, as well as 5623 (4688 nn × np plus 935 hk × hk) constituted linkage groups on the QG map with a total genetic distance of 1687.33 cM and 0.30 cM per marker (Table 3; Additional file 2: Figure S1; Additional file 3: Table S2).

**Table 3:** Profile of parental and integrated maps. (HC, ‘Honeycrisp’; QG, ‘Qinguan’)

Linkage group	HC map			QG map			Integrated map		
	Number of markers	Genetic distance (cM)	Average distance between markers (cM)	Number of markers	Genetic distance (cM)	Average distance between markers (cM)	Number of markers	Genetic distance (cM)	Average distance between markers (cM)
1	256	136.63	0.53	260	118.66	0.46	503	162.27	0.32
2	268	91.85	0.34	296	119.23	0.40	540	168.33	0.31
3	261	112.98	0.43	320	109.90	0.34	521	154.49	0.30
4	270	110.32	0.41	324	93.87	0.29	560	133.28	0.24
5	366	65.44	0.18	413	109.02	0.26	731	121.69	0.17
6	497	75.55	0.15	205	97.34	0.47	661	117.63	0.18
7	292	140.87	0.48	430	93.26	0.22	638	160.34	0.25
8	367	83.40	0.23	174	114.18	0.66	496	124.67	0.25
9	318	80.07	0.25	322	97.55	0.30	590	115.99	0.20
10	416	94.91	0.23	162	92.27	0.57	547	128.87	0.24
11	408	102.26	0.25	321	72.32	0.23	650	120.57	0.19
12	226	111.39	0.49	228	109.44	0.48	403	147.47	0.37
13	257	159.75	0.62	819	94.41	0.12	1057	161.80	0.15
14	225	95.35	0.42	368	89.86	0.24	497	124.50	0.25
15	235	133.88	0.57	377	83.05	0.22	574	179.97	0.31
16	423	112.27	0.27	345	90.09	0.26	690	170.80	0.25
17	266	130.70	0.49	259	102.89	0.40	514	137.85	0.27
Total	5351	1837.61		5623	1687.33		10172	2430.52	
Average			0.34			0.30			0.24

### Phenotyping of δ^13^C (‰) in HC × QG population under two irrigation regimes during two-year experiment

We determined the δ^13^C of two parent cultivars and their progenies for two watering treatments in 2014 and 2015 (Table 4). The δ^13^C was always lower for HC than for QG, indicating that the drought-tolerant trait had a strong genetic component. Moreover, the range in variation for δ^13^C in the segregating population always exceeded the phenotypic values of parents, suggesting that transgressive inheritance was continuous and was not influenced by either treatment or year. For all samples- parents and progenies-the induction of drought stress had a strong impact on δ^13^C.

**Table 4:** Means and range in variation of δ^13^C (‰) in HC×QG population under two irrigation regimes in two consecutive years of treatment

		2014		2015	
		Well-watered (±SD)	Drought (±SD)	Well-watered (±SD)	Drought (±SD)
Honeycrisp		-28.081±0.037	-27.322±0.040	-28.160±0.042	-27.164±0.037
Qinguan		-27.581±0.041	-26.591±0.019	-26.940±0.046	-26.203±0.033
Progeny	Average	-27.477±0.446	-26.769±0.334	-27.465±0.396	-26.757±0.317
	Max	-26.107±0.025	-25.983±0.048	-26.549±0.035	-25.658±0.049
	Min	-28.449±0.031	-27.973±0.026	-28.503±0.028	-27.867±0.041

**Fig. 1:**
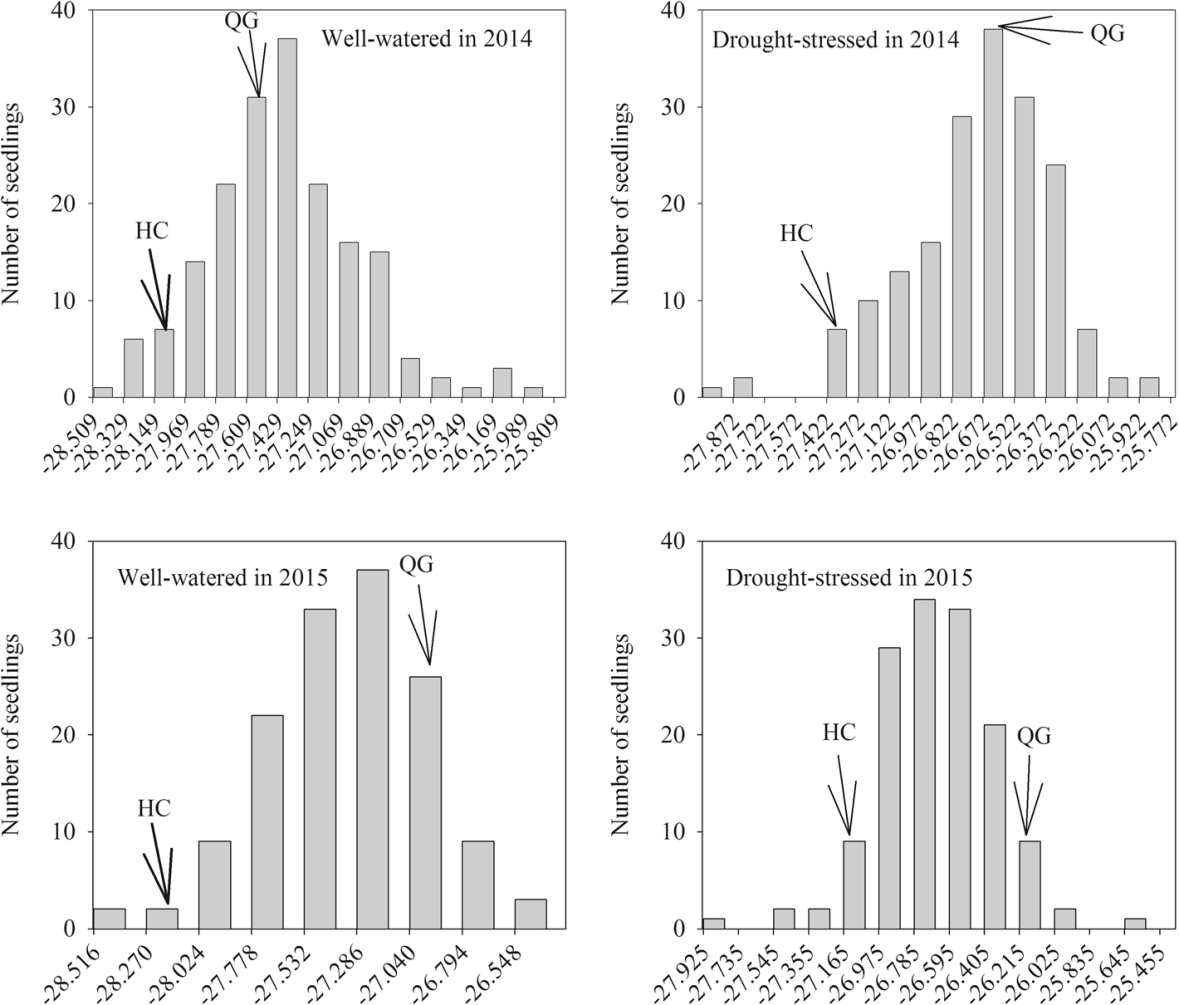
Frequency distribution of δ^13^C (%) for of progeny (HC × QG) under well-watered and drought-stress conditions in 2014 and 2015. Phenotypic values of parents are indicated by arrows

The frequency distributions of δ^13^C for the HC × QG mapping population were continuous and near-normal for both the well-watered and drought-stressed treatments over the two test years (Fig. 1). This suggested that the response was quantitative and controlled by multiple loci. Under a water deficit, the moving δ^13^C distribution toward a higher value indicated the promotion of drought stress to the trait (Fig. 1; Table 4).

### Detection of QTLs for δ^13^C under two irrigation regimes over two years

**Fig. 2:**
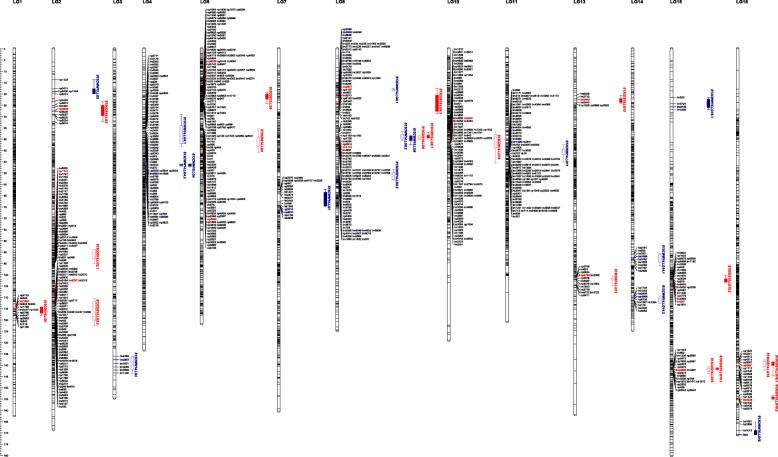
QTLs for δ^13^C under two irrigation regimes over consecutive years, as identified in HC × QG mapping population. LG means linkage group. QTLs are indicated by boxes (1-LOD interval) and extended lines (2-LOD intervals). Solid and grid boxes represent QTLs detected in 2014 and 2015, respectively; QTLs detected under well-watered and drought-stress conditions are represented with blue and red colors, respectively. Genetic distance is shown in centimorgan (cM) on left side

We detected 33 QTLs for δ^13^C of apple that were mapped over 13 LGs of HC × QG map (Table 5; Fig. 2). These loci included 9 and 8 under well-watered and drought-stressed conditions, respectively, in 2014, and 6 (well-watered condition) and 10 (drought-stressed condition) in 2015.

**Table 5:** QTLs for δ^13^C under two irrigation regimes

Treatment	QTL^a^	LG^b^	Interval (cM)	Peak position (cM)	Marker	LOD	Variance (%)
Well-watered in 2014	δ13CWW14.LG3	LG3	136.03-143.49	137.63	lm2857	3.70	8.8
	δ13CWW14.LG4.1	LG4	29.09-43.13	40.25	lm5833	3.64	8.7
	**δ13CWW14.LG4.2**	LG4	51.03-51.93	51.40	np763	3.03	7.3
	δ13CWW14.LG8.1	LG8	17.46-18.68	17.87	lm3625	4.59	10.8
	**δ13CWW14.LG8.2**	LG8	36.54-40.60	39.38	lm1712	4.74	11.1
	δ13CWW14.LG8.3	LG8	53.52-58.14	57.68	lm4346	3.64	8.7
	δ13CWW14.LG11	LG11	42.36-50.08	45.77	lm1426	3.08	7.4
	δ13CWW14.LG14.1	LG14	90.53-95.77	92.58	hk1099	6.10	17.8
	δ13CWW14.LG14.2	LG14	109.28-112.46	110.69	lm5786	3.75	11.4
Well-watered in 2015	δ13CWW15.LG2	LG2	13.65-19.89	19.00	np1104	3.15	9.6
	**δ13CWW15.LG4**	LG4	51.03-52.23	51.40	np763	3.05	9.4
	δ13CWW15.LG7	LG7	62.20-69.69	67.47	hk2442	5.95	17.4
	**δ13CWW15.LG8**	LG8	38.22-42.49	39.38	lm1712	3.19	9.8
	δ13CWW15.LG15.1	LG15	21.54-26.91	25.71	lm2143	6.14	17.9
	δ13CWW15.LG16	LG16	165.18-170.8	170.80	hk5	4.28	12.9
Drought-stressed in 2014	δ13CDS14.LG2.1	LG2	88.81-94.89	89.20	lm1741	5.30	11.9
	δ13CDS14.LG2.2	LG2	110.37-122.48	112.11	lm5787	3.70	8.5
	δ13CDS14.LG5	LG5	35.34-45.77	43.09	np5995	4.00	9.2
	**δ13CDS14.LG8**	LG8	38.22-40.21	39.38	lm1712	3.62	8.3
	δ13CDS14.LG10	LG10	36.29-50.51	40.28	lm2441	3.77	8.6
	δ13CDS14.LG13	LG13	100.61-104.78	101.42	np2705	3.13	7.2
	**δ13CDS14.LG15**	LG15	140.67-143.58	141.57	np2623	3.29	7.6
	**δ13CDS14.LG16**	LG16	137.6-142.11	139.09	np2691	3.45	7.9
Drought-stressed in 2015	δ13CDS15.LG1	LG1	114.32-117.91	114.92	np1493	3.54	10.8
	δ13CDS15.LG2	LG2	23.42-32.31	27.52	np6039	4.20	12.6
	δ13CDS15.LG5	LG5	19.31-24.34	21.88	np6056	4.08	12.3
	**δ13CDS15.LG8.1**	LG8	36.95-39.58	39.38	lm1712	4.28	9.8
	δ13CDS15.LG8.2	LG8	17.46-28.81	24.67	lm2877	3.38	10.3
	δ13CDS15.LG13	LG13	20.56-24.25	22.68	lm4452	3.21	9.8
	**δ13CDS15.LG15.1**	LG15	141.24-142.03	141.57	np2623	4.02	12.1
	δ13CDS15.LG15.2	LG15	100.18-103.34	103.24	lm637	3.29	7.6
	**δ13CDS15.LG16.1**	LG16	138.3-144.73	139.09	np2691	4.02	12.1
	δ13CDS15.LG16.2	LG16	153.56-155.33	154.71	hk3338	4.79	14.3

^a^ Names of QTLs that were stable between years under the same treatment were shown with boldface. ^b^The linkage groups of integrated map was used to detect the QTLs

One QTL cluster was detected on LG8, which had four significant QTLs under different irrigation regimes in two years. The clustering QTLs on LG8 involved the same marker lm1712, which was close to their LOD peaks. Besides, three regions that encompassed stable QTLs were found on LG4, LG15 and LG16 under the same irrigation regimes in the two years (Table 5; Fig. 2). Among them, δ^13^CWW14.LG4.2 and δ^13^CWW15.LG4 were overlapped and shared the same marker np763 with peak LOD scores. δ13CDS14.LG15 and δ13CDS15.LG15.1 located the same region with the same marker np2623 close to their LOD peaks. δ13CDS14.LG16 and δ13CDS15.LG16.1 were co-located with the same marker np2691 closest to their QTL peaks. δ13CWW14.LG8.1 and δ13CDS15.LG8.2 were also mapped in the close region, but they carried different SNPs with peak LOD scores. The co-locations of these QTLs suggested that existence of the same genetic determinants involved in them.

In 2014, three stable QTLs on LG8, LG15 and LG16 for δ^13^C under drought stress condition explained 8.3, 7.6 and 7.9% of phenotypic variation, with respective LOD scores of 3.62, 3.29 and 3.45 (Table 5). The following year, the variances of these QTLs accounted for 9.8, 12.1 and 12.1%, with LOD scores of 3.38, 4.02 and 4.02, respectively (Table 5).

### Validation of QTLs for δ^13^C under drought-stress based on KASP assay-based SNP marker genotyping

**Fig. 3:**
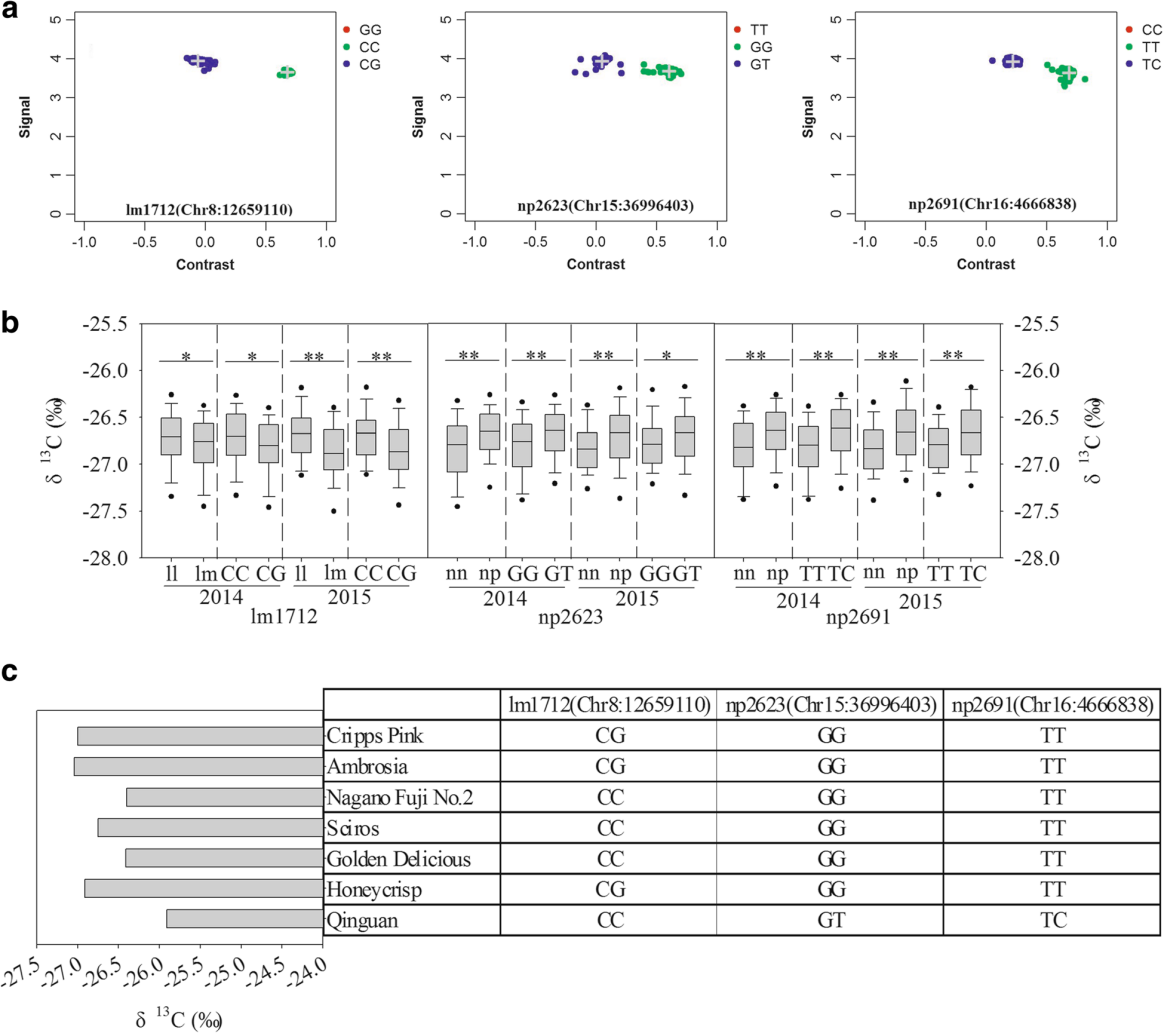
Validation of QTLs for δ^13^C under drought-stressed conditions, based on genotyping using KASP assay-based SNP markers. **a** genotype plot on 3 positions (lm1712, np2623, and np2691) for KASP platform. **b** phenotypic distribution of δ^13^C measured in HC × QG segregating population under drought-stressed conditions, based on 3 stable positions (lm1712, np2623, and np2691). lm, ll, nn, and np represent genotypes detected by RAD-SNP marker genotyping. Base pairs represent genotypes detected by KASP array. Significance levels of differences at *p* < 0.05 and *p* < 0.01 are indicated with “*” and “**”, respectively. **c** genotypes of 7 commercial apple cultivars on 3 stable positions (lm1712, np2623, and np2691), as detected by KASP assay. Phenotypic values of δ^13^C in several cultivars under drought-stressed conditions were reported by Liu et al. [28]

The SNP markers (lm1712, np2623, and np2691) closest to the peaks of three QTLs that proved stable for δ^13^C across years under drought stress were putatively located to Chromosome 8 (Chr8): 12659110, Chr15: 36996403, and Chr16: 4666838 according to Apple Genome v1.0. Our comparison of results from KASP (Fig. 3a) and RADseq genotyping of the same progenies revealed highly consistent rates for those seedlings, i.e., 89.64, 92.23, and 92.23% for markers lm1712, np2623, and np2691, respectively (Table 6).

**Table 6:** Consistency of individuals within groups genotyped by RAD-SNP and KASP

Position	Genotype	RAD-SNP	KASP	Consistent amount	Consistent rate
Chr8: 12659110	lm/CG	94	88	80	89.64%
	ll/CC	99	105	93	
Chr15: 36996403	nn/GG	103	117	102	92.23%
	np/GT	90	76	76	
Chr16: 4666838	nn/TT	113	122	108	92.23%
	np/TC	80	71	70	

We also investigated statistical differences in average phenotypic values under a water deficit between individuals genotyped by those two methods, and found similar patterns (Fig. 3b). For np2623 and np2691, individuals carrying the heterozygous genotype GT (np) and TC (np) showed higher phenotypic values, whereas, for lm1712, the phenotypic values were significantly higher for individuals carrying the homozygous genotype CC (ll).

Besides our parental cultivars, we used these three SNP markers to genotype five other commercial apple cultivars (Fig. 3c). While ‘Qinguan’ was heterozygous for np2623 and np2961, with ‘GC’ and ‘TC’ genotypes, respectively. All other cultivars were homozygosis (‘GG’ and ‘TT’) at those two positions. For lm1712, ‘Cripps Pink’, ‘Ambrosia’, and ‘Honeycrisp’ showed heterozygosis with the ‘CG’ genotype, while the other cultivars displayed homozygous ‘CC’. We analyzed their δ^13^C phenotypic values we have reported previously [28] (Fig. 3c) and noted obvious differences among these cultivars. The highest δ^13^C (− 25.91‰) was observed for ‘Qinguan’. ‘Ambrosia’, ‘Cripps Pink’, and ‘Honeycrisp’ all had lower values, i.e., − 27.04‰, − 27.00‰, and − 26.91‰, respectively. Moderate levels were found for ‘Golden Delicious’ (− 26.41‰) and ‘Nagano Fuji No. 2’ (− 26.40‰). When all of these genotyping results were considered, we were able to distinguish the loci of np2623 and np2691 for ‘Qinguan’ (high-value, heterozygous genotype) from a group of mixed genotypes, while the loci of lm1712 could distinguish among ‘Ambrosia’, ‘Cripps Pink’, and ‘Honeycrisp’, all of which had lower phenotypic values (heterozygous genotype).

### Potential candidate genes involved in determining apple δ^13^C under drought stress

**Fig. 4:**
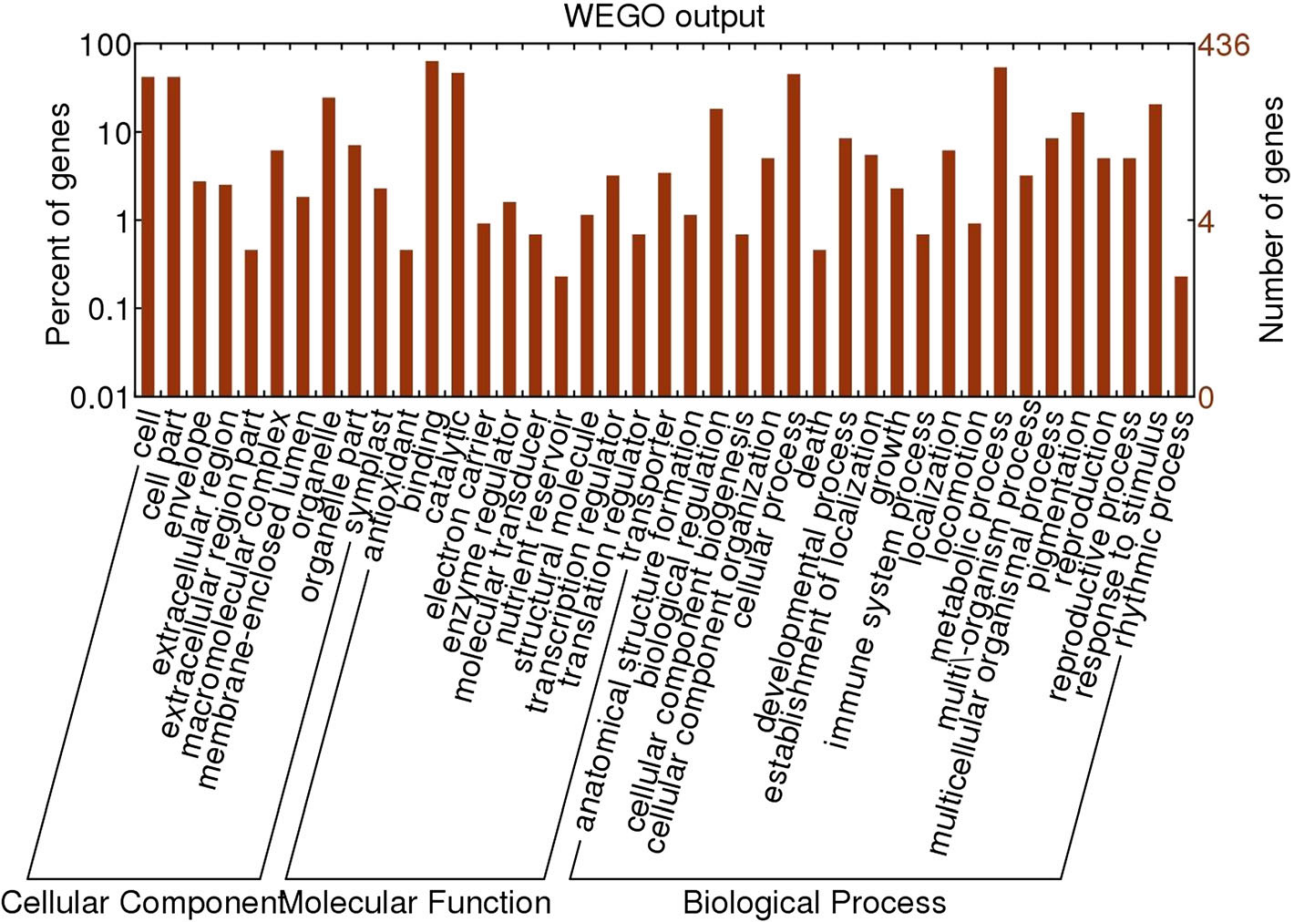
WEGO histogram for 435 annotated genes of apple in 3 targeted QTL intervals of δ^13^C under drought stress. Right-hand and left-hand y-axes, number and corresponding percentage of genes, respectively, within specific category

Genes involved in δ^13^C of drought-stressed apple were investigated according to the alignment of physical positions in the ‘Golden Delicious’ genome that showed three overlapped intervals of QTLs. The three regions were 12.58 to 13.17 Mb on Chr8 (38.22–39.58 cM of LG8), 34.70 to 37.80 Mb of Chr15 (141.24–142.03 cM of LG15), and 3.68 to 4.87 Mb of Chr16 (138.30–142.11 cM of LG16), which contained 106, 254, and 167 genes, respectively (Additional file 4: Table S3). The GO annotations for 435 of them were uploaded to WEGO (Additional file 5: Table S4; Fig. 4). Based on their more specific roles in molecular functions, biological processes, or cellular components, 166 genes were prioritized in categories that included substance metabolism and modification, response to stress or hormone, signaling, transport, regulation of RNA transcription, photosynthesis, respiration, and regulation of organ development. We also examined another 92 genes without GO annotations. All candidate genes are listed in Additional file 6: Table S5.

### Expression profiling of *Arabidopsis* orthologs of candidate genes

**Fig. 5:**
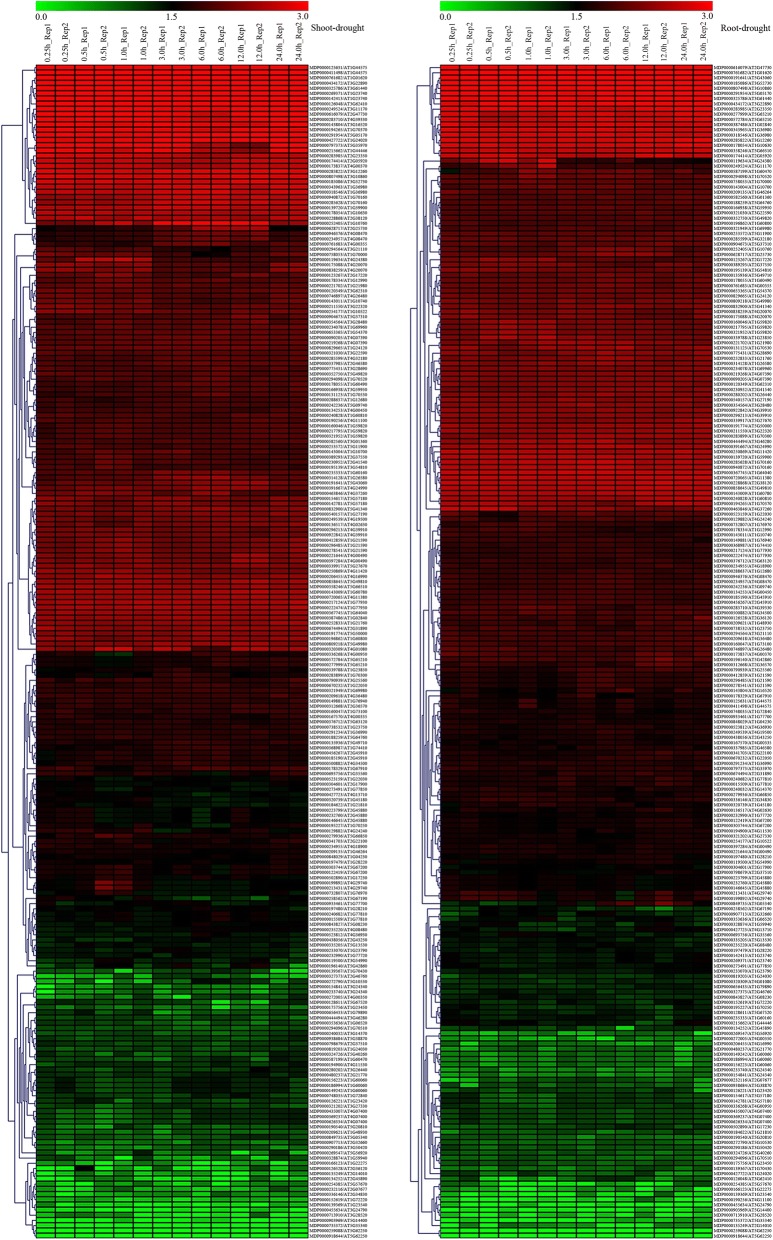
Expression analysis of *Arabidopsis* orthologs of candidate genes in apple. Patterns found for orthologs in shoot (left) and root (right) of *Arabidopsis* under drought stress. Color gradient from green to red represents range in expression from lower to higher

Using protein sequences of the 258 candidate genes (166 genes possessing prioritized GO annotations and 92 genes without GO annotations) for apple δ^13^C under drought stress as queries, we performed a local blast to find their homologous genes in *Arabidopsis*. Then we extracted the transcriptome data of their homologous genes in *Arabidopsis* in response to drought stress (GSE5624) from the NCBI database (http://www.ncbi.nlm.nih.gov) (Additional file 7: Table S6). Among these candidates, five genes did not hit any homologous genes and 27 lacked expression data (Additional file 7: Table S6). After data extraction for the remaining genes, the expression profiles of the orthologs in *Arabidopsis* were displayed in the form of hierarchical clustering heatmaps (Fig. 5). The expression of many genes did not change significantly in *Arabidopsis* under drought-stressed treatment (Fig. 5; Additional file 7: Table S6). However, 132 and 35 differentially expressed genes were detected in the *Arabidopsis* shoots and roots, respectively (Additional file 7: Table S6). This suggested that our stable QTLs might contain drought-responsive genes. The results also confirmed that those genetic regions have potential value when searching for genes that regulate WUE under drought stress condition.

Many identified differentially expressed genes were involved in protein metabolism and modification (Additional file 5: Table S4, Additional file 6: Table S5). Kinase genes constituted the main, including MDP0000946376 and MDP0000235220, which encode the mitogen-activated protein kinase kinase kinase; MDP0000502890, for receptor-like kinase protein FLORAL ORGAN NUMBER1; and six BRASSINOSTEROID INSENSITIVE 1-associated receptor kinase 1 genes (MDP0000195227, MDP0000907713, MDP0000540157, MDP0000131123, MDP0000500882 and MDP0000122419) (Additional file 6: Table S5, Additional file 7: Table S6). We also found phosphatase 2A catalytic subunit alpha isoform gene, MDP0000234078; and two cysteine protease genes, MDP0000166938 and MDP0000196140. Several drought-responsive transcriptional factors (TFs) genes were also revealed in *Arabidopsis*. Their orthologs in apple included MDP0000463846 (*GL1*), MDP0000126221 (*YABBY 4*), MDP0000758053 (*MYB1R1*), MDP0000232116 (*ARF*), MDP0000713910 (*bHLH95*), MDP0000258562 (*RAP2–9*), MDP0000129882 (*WRKY 7*), and MDP0000444494 (*ERF020*). Homologous genes related to hormone metabolism or regulation were MDP0000199892, MDP0000211550, and MDP0000213431, encoding cytokinin dehydrogenase; MDP0000228868, for auxin transporter-like protein; and MDP0000230952, for gibberellin-regulated protein. Two orthologs were involved in chlorophyll metabolism: MDP0000215662 and MDP0000735372. Other apple genes with drought-responsive homologs in *Arabidopsis* showed important roles in substance metabolism, e.g., MDP0000480237, encoding cellulose synthase A catalytic subunit; and two beta-amylase genes, MDP0000221644 and MDP0000397284. Our screening also resulted in the identification of structural genes that respond to drought in *Arabidopsis*. Their orthologs in apple included MDP0000427722 (MLP-like protein 423), MDP0000587199 (galactinol synthase 1), and MDP0000809218 (inhibitor response 1-like protein).

### Prediction of interaction network for *Arabidopsis* orthologs of candidate genes

**Fig. 6:**
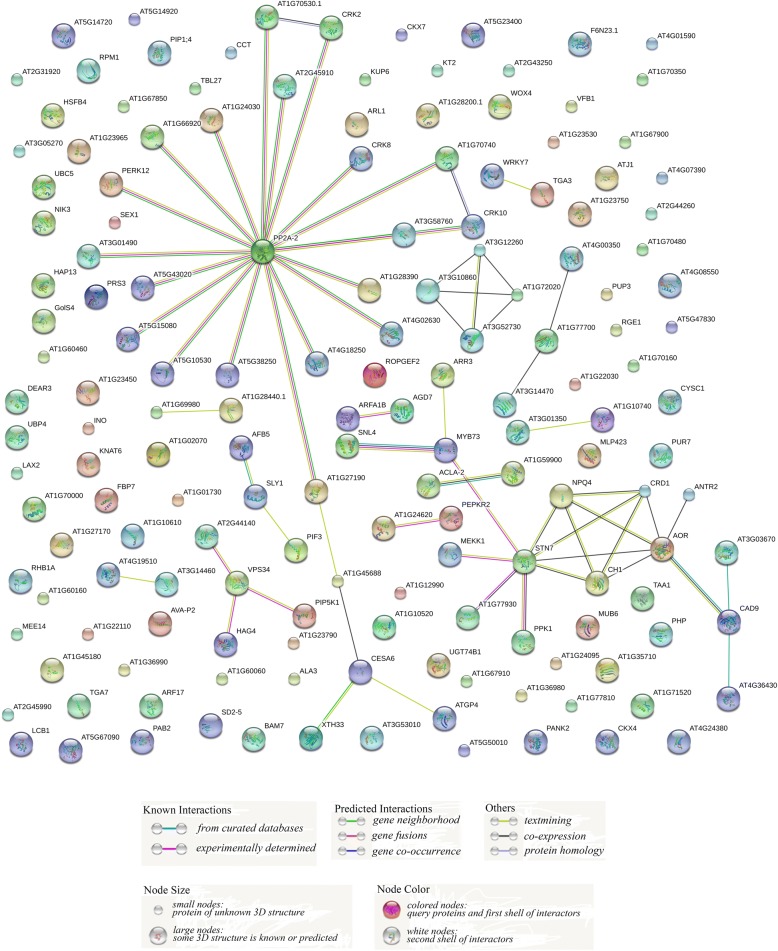
Predicted interaction network for proteins encoded by candidate genes based on their orthologs in *Arabidopsis*, as acquired via STRING. Large and small nodes in networks represent proteins with or without known 3D structures, respectively. Colored nodes represent the query proteins, while white nodes represent the interacting proteins provided by STRING. Protein interactions are judged on the basis of known curated databases and experiments, predicted gene neighborhood, fusions and co-occurrence, or other types of strategies (text mining, co-expression and protein homology), which are represented by different colored lines between nodes

The STRING database (http://string-db.org), an online prediction tool for protein-protein interactions, was employed to develop protein interaction networks for candidate genes involved in controlling apple δ^13^C under drought stress. Using the protein sequences of these 258 candidate genes as queries, we predicted protein interactions (Fig. 6; Additional file 8: Table S7) and obtained a large umbrella-type network (Fig. 6) via STRING. The central member of this network was protein phosphatase 2A-2 (PP2A-2), the ortholog of MDP0000234078. Proteins interacting with PP2A-2 included a U-box domain-containing protein 33 (AT2G45910, the ortholog of MDP0000456267) and several protein kinases, e.g., CRK2 (ortholog of MDP0000294098) and PERK12 (ortholog of MDP0000139369). The protein cellulose synthase 6 (CESA6), which was associated with PP2A-2 via AT1G27190 (a putative kinase), and AT1G45688 (an uncharacterized protein), were positioned on one branch of the network. At the terminal of that branch, CESA6 (the ortholog of MDP0000480237 and MDP0000291954) interacted with xyloglucan:xyloglucosyl transferase 33 (XTH33, ortholog of MDP0000233177) and membrane-anchored ubiquitin-fold protein (ATGP4, ortholog of MDP0000391667).

Another large network contained several important proteins related to photosynthesis, such as serine/threonine protein kinase STN7 (ortholog of MDP0000674494), nonphotochemical quenching 4 (NPQ4, common ortholog of MDP0000125631 and MDP0000411498), and two catalyzing enzymes of chlorophyll biosynthesis, i.e., copper response defect 1 (CRD 1, ortholog of MDP0000735372) and CHLORINA 1 (CH1, ortholog of MDP0000215662) (Fig. 6). In addition, STN7 interacted with MYB73 (ortholog of MDP0000463846), kinase MEKK1 (common ortholog of MDP0000234957, MDP0000235220, and MDP0000946376), kinase PPK1 (ortholog of MDP0000232990), alkenal/one oxidoreductase AOR (ortholog of MDP0000142413), and chaperone DnaJ-domain-containing protein AT1G77930 (ortholog of MDP0000217124). We also detected dyadic interactions among NPQ4, STN7, CH1, CRD1, and AOR in that network. AT3G12260, AT3G52730, AT3G10860, and AT1G72020 (orthologs of MDP0000285822, MDP0000185086, MDP0000807498 and MDP0000628717, respectively) interacted with each other in a square network (Fig. 6). Because AT3G12260 was annotated as NADH dehydrogenase (ubiquinone) 1 alpha subcomplex 6, which is the accessory subunit of the mitochondrial membrane respiratory chain NADH dehydrogenase (complex I), and AT3G52730 was annotated as ubiquinol-cytochrome c reductase subunit 9, we could conclude that the genes for respiration are involved in these QTLs.

Several simple interactions were also detected (Fig. 6). For example, vacuolar protein sorting 34 (VPS34), the ortholog of MDP0000178055, was associated with proteins PIP5K1, HAG4, and AT2G44140 (orthologs of MDP0000221702, MDP0000242236, and MDP0000166938, respectively). As an ortholog of MDP0000126528, SLY1 might positively regulate the gibberellin signaling pathway, interacting with auxin F-box protein 5 (AFB5) and PIF3, which are orthologs of MDP0000809218 and MDP0000523812, respectively (Fig. 6). All of these proteins that constitute interaction networks might play more important roles, making them preferential candidates.

### Expression analysis of selected candidate genes for responses to long-term drought stress

**Fig. 7:**
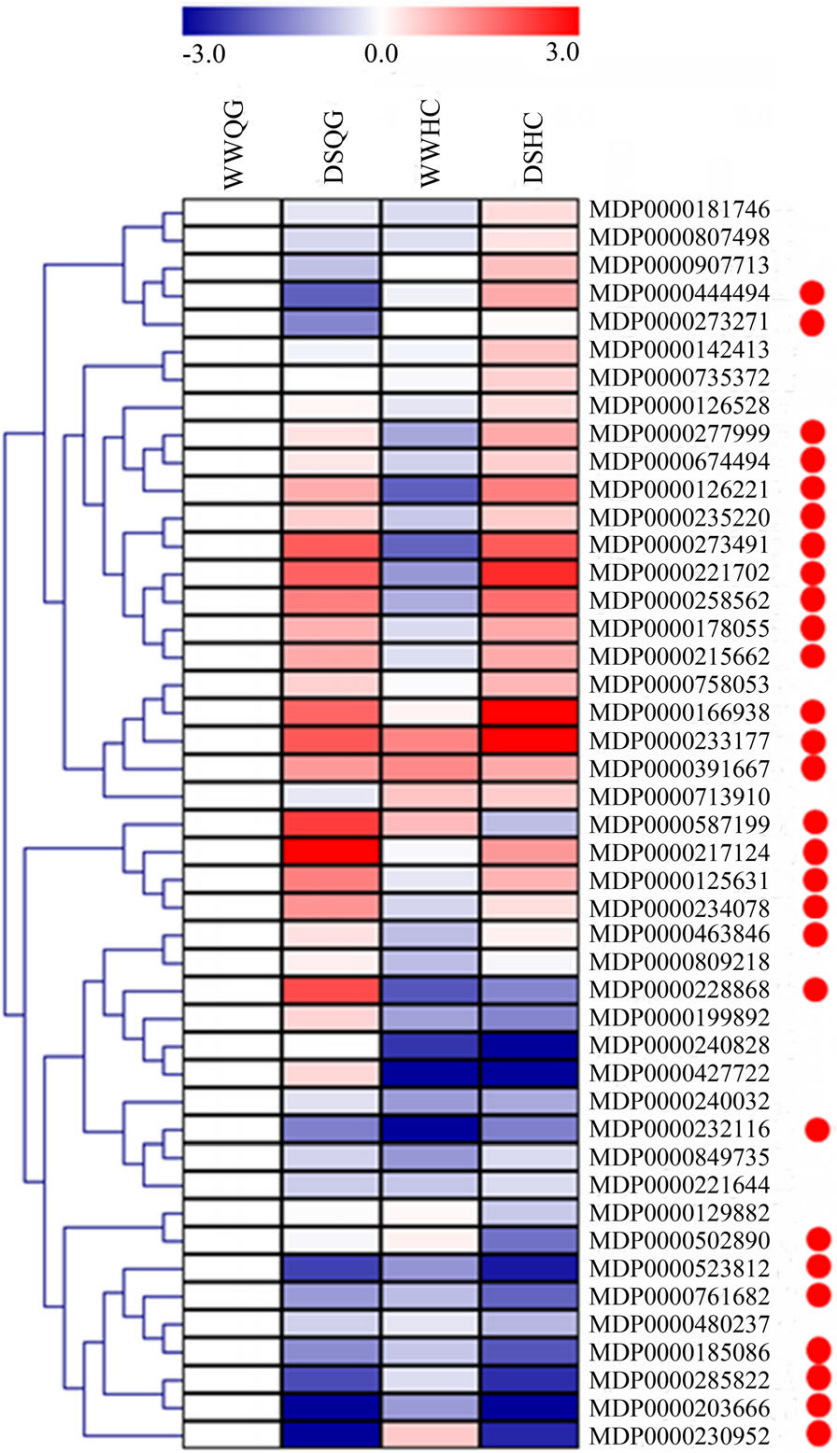
Expression analysis via qRT-PCR of select genes in ‘Qinguan’ (QG) and ‘Honeycrisp’ (HC) in response to long-term drought stress. ‘WWQG’, ‘DSQG’, ‘WWHC’ and ‘DSHC’ indicate the well-watered ‘Qinguan’, drought-stressed ‘Qinguan’, well-watered ‘Honeycrisp’ and drought-stressed ‘Honeycrisp’, respectively. Gene expression profiles are shown using hierarchical clustering method. Gene IDs are listed on the right. Candidate gene is marked with red spot. Color gradient from blue to red represents range in expression from lower to higher

For preliminary identification of important regulators of apple WUE, we used qRT-PCR to study the expression patterns of 45 genes in ‘Qinguan’ and ‘Honeycrisp’ plants exposed to long-term drought stress (Fig. 7; Additional file 9: Table S8). These genes were selected because of either the marked responses of their *Arabidopsis* orthologs or their predicted interaction effects. We also investigated genes that, although they did not fit either of those criteria, might still play roles in regulating WUE, based on their functional descriptions (Additional file 5: Table S4; Additional file 6: Table S5). These genes included MDP0000761682 (*PIP1*), MDP0000181746 (*bHLH145*), MDP0000203666 (*WOX4*), MDP0000277999 (*bZIP1*) and MDP0000273491 (*ARF17*).

Among all genes selected for this comparative analysis, 28 exhibited not only significant responses to drought stress (*p* < 0.05) in at least one cultivar but also more than two-fold changes in expression levels. The functional descriptions of these prioritized genes (marked by red-spots in Fig. 7; bolded listings in Additional file 9: Table S8) showed that they might be involved in multiple physiological processes associated with WUE under drought stress condition, i.e., signaling (MDP0000234078 and MDP0000178055), photosynthesis (MDP0000125631, MDP0000674494, and MDP0000215662), response to stresses (MDP0000587199, MDP0000217124, and MDP0000273271), carbohydrate metabolism (MDP0000233177 and MDP0000480237), protein metabolism and modification (MDP0000235220, MDP0000166938, and MDP0000502890), hormone metabolism and transport (MDP0000228868 and MDP0000230952), transport (MDP0000761682), respiration (MDP0000285822 and MDP0000185086), transcriptional regulation (MDP0000273491, MDP0000258562, MDP0000463846, MDP0000523812, MDP0000203666, MDP0000277999, and MDP0000444494), and development regulation (MDP0000126221, MDP0000232116, and MDP0000221702) (Additional file 6: Table S5). Some genes of them took part in more than one process, such as MDP0000234078, MDP0000587199, MDP0000463846, MDP0000178055 and so on (Additional file 6: Table S5). Because of the marked responses of these 28 genes to drought stress, we considered them potential candidates for examining the WUE mechanism in drought-stressed apple.

## Discussion

The stability of δ^13^C and its high correlation with long-term WUE provides a more reliable and higher throughput indicator to determine the complex relationship between carbon fixation and water use by annual crops [44, 45] as well as by tree species, e.g., *Pseudotsuga menziesii* [46], *Pinus pinaster* [47], *Quercus robur* [26, 48], and *Malus* spp. [27, 28]. The genetic determinism of plant WUE, as estimated by δ^13^C responses to water deficits, has been widely studied in various species of perennial plants [47–52]. Δ^13^C (the negatively correlated parameter of δ^13^C) in apple leaf exhibited a significantly negative correlation with WUE in the level of fruit, leaf + fruit or total biomass, indicating that assessing this parameter was an cost-efficient method to evaluate long-term WUE in apple [29]. Based on these, we selected δ^13^C as the indicator of WUE in apple. As far as we know, our report is the first to focus on the identification of QTLs for δ^13^C in apple under drought stress condition.

This large number of QTLs explaining low percentages of the phenotypic variance confirms the hypothesis of polygenic inheritance of apple δ^13^C [51, 52]. Thus, using δ^13^C in C3 plants as a measure of isotopic composition (^13^C and ^12^C) in plants, compared with levels in the atmosphere, would mean that all of those factors that influence carbon fixation and metabolism can affect the δ^13^C value of a plant [53]. Because δ^13^C is closely correlated with whole-plant WUE, the QTLs for δ^13^C detected in our study could be genetic regulators of long-term WUE in apple. Two hotspot regions were detected on LG8, regardless of irrigation regime or year tested. The corresponding physical position of that hotspot on LG8 was approximately 12.58 to 13.17 Mb for Chr8 near marker CH02g09 in the contig MDC002525.346 (14.30–14.35 Mb) on Chr8, which is involved in QTLs for traits associated with gas exchange [21], xylem conductance [54], and fruit production [55], as well as two visual variables related to canopy structure/biomass production and crop nitrogen status [22]. Lauri et al. suggested that this proximity was due to the increase in capacity to transport water and carbohydrates to developing organs [54]. Therefore, the QTLs for traits related to those processes demonstrate co-localization. It is possible that such co-localizations of QTL groups are a consequence of pleiotropy by functionally related genes [56].

Apple is a large, woody perennial with a long juvenile period. Such characteristics mean that this organism is ill-suited for conventional analyses that rely upon high-throughput phenotypic evaluations of complex traits. Therefore, MAS provides an efficient, alternative approach for pre-selecting targeted individuals for breeding programs. In addition to SSR markers, which are used when evaluating seedlings for disease susceptibility/resistance [57] or high texture performance [58], SNP-based markers are gradually being developed as potential candidates for MAS. These include the development of SNP-based *MdAAT1*-specific marker, which can distinguish cultivars with medium-high ester concentrations in their fruits from cultivars with low concentrations [16]. Seven SNPs closest to QTLs for the concentrations of soluble sugars and acids in apple have indicated candidate markers for MAS that can help improve breeding programs for enhanced fruit quality [17]. The uniplex KASP platform, now a standard, cost-effective technology with scalable flexibility and a lower error rate, is widely applied in SNP genotyping [59, 60]. The transferability of SNPs in Illumina assay to KASP with lower error rate has been verified [59]. Based on our mapping of QTLs for δ^13^C, we developed three stable loci into KASP assay-based SNP markers. Genotyping the same population through this methods produced results similar to those achieved with the RADseq-based SNPs. Those markers were able to distinguish different phenotypic values among several commercial cultivars. These findings suggested that the potentials of these SNPs to distinguish the genotypes with different δ^13^C and also validated the reliability of the QTLs involving these SNPs for δ^13^C under drought-stressed treatment. This outcome demonstrates the potential that these markers have for future MAS of higher WUE in apple under drought stress.

In this study, 28 candidate genes showed significant responses to drought stress or differential expression patterns between ‘Qinguan’ and ‘Honeycrisp’. They are involved in photoprotection, signal transduction, metabolism and transport, and transcriptional regulation. All are possibly connected with WUE in apple. Proteins encoded by MDP0000125631 (*NPQ4*) that were identified here were also detected in a previous proteome analysis that showed differential expression between drought-stressed and well-watered ‘Qinguan’ (high-WUE) or ‘Fuji’ (low-WUE) apple [8]. Zhou et al. revealed that the increment in NPQ over long-term drought is obviously higher in ‘Qinguan’ and concluded that the former has greater ability to avoid photodamage [8]. Likewise, MDP0000125631 (NPQ4) was significantly up-regulated in ‘Qinguan’ rather than ‘Fuji’ under long-term drought, again suggesting that NPQ is an important mechanism by which apple trees respond to drought [8]. We examined the expression of MDP0000125631, which showed up-regulation in both cultivars under drought. STN7 is a critical serine/threonine protein kinase required for state transition that is achieved through phosphorylation of the outer antennae of light-harvesting complex II (LHCII) [61]. We predicted an interaction between STN7 and NPQ4. The state transition of the former has a central role in responses to environmental changes, allowing plants to adjust to changing levels of irradiance by redistributing light excitation energy between Photosystem II (PSII) and PSI [62]. We also noted that the patterns of drought response were similar between MDP0000674494 (*STN7*) and MDP0000125631 in our two tested cultivars. Another protein that interacts with STN7 is DnaJ. Our results showed that drought stress significantly induced apple DnaJ gene MDP0000217124 in both drought-stressed cultivars, especially in ‘Qinguan’. Heterologous expression of tomato chloroplast-targeted DnaJ protein *LeCDJ2* in tobacco significantly enhances the drought tolerance, and reduces photoinhibition by maintaining the stability of PSII D1 protein [63]. The coincidental up-regulation of MDP0000674494, MDP0000217124 and MDP0000125631 which were encompassed in drought-specific QTLs on LG15 and LG16 indicates that not only *DnaJ* probably functions with *STN7*, but also confirms that photoprotection is an important mechanism when apple respond to drought stress and regulate WUE under such stress.

A complete MAPK signaling pathway, AtMEKK1-AtMKK2/AtMKK1-AtMPK4, was identified in *Arabidopsis*, where it was shown to transfer signals of drought and wounding [64]. des Marais et al. demonstrated the effect of MPK12 on WUE in *Arabidopsis* [11]. We noted that the expression of MDP0000235220 (*MAPKKK*) in drought-specific QTL on LG15 in both cultivars was up-regulated by drought, suggesting its stress-responsiveness. We found its homolog MEKK1 in *Arabidopsis* interacts with STN7. And, the patterns of expression patterns were similar for MDP0000235220 and MDP0000674494 in both tested cultivars exposed to long-term stress. These imply an inner connection between drought signaling and photoprotection in apple via MAPK cascades. Screening revealed another protein phosphatase gene, MDP0000234078 (*PP2A-2*). PP2A-2 is a negative regulator of ABA signaling in *Arabidopsis* [65]. Our expression data showed that MDP0000234078 in drought-specific QTL on LG16 was obviously induced by drought in both cultivars, implying that it is involved in the response of apple to long-term drought. However, the regulatory mechanism of this gene might be more complicated because our interaction network indicated that it is associated with numerous kinases in the confidence intervals.

An interaction was predicted between XTH33 and CESA6. The former participates in the processes of cell wall modification and organ elongation [66], while the latter is a necessary synthetase for the formation of primary walls in *Arabidopsis* [67]. MDP0000233177 (*XTH33*) was obviously up-regulated by long-term drought, especially in ‘Honeycrisp’. MDP0000480237 (*CESA6*) showed similar down-regulation pattern in both cultivars under water deficit. These contrasting responses might influence polysaccharide metabolism and cell wall formation in apple under drought stress, which could be related to WUE because of its effect on carbon distribution.

Under drought stress, expression of MDP0000228868 for auxin transport was induced in ‘Qinguan’ but remained relatively stable, albeit at a lower level, in ‘Honeycrisp’. We inferred from this that the significant enhancement of MDP0000228868 under drought helps promote auxin transport and maintains regular growth in stressed ‘Qinguan’.

Among the drought-responsive TFs, ARF17 (MDP0000273491) was identified as a differentially expressed protein in drought-stressed ‘Fuji’ apple [8]. Besides *ARF17*, we also identified significantly drought-responsive *GL1* (MDP0000463846), *YABBY4* (MDP0000126221), *RAP2–9* (MDP0000258562), *bZIP-1* (MDP0000277999), *ERF020* (MDP0000444494), *WOX4* (MDP0000203666), and *bHLH056* (MDP0000523812). Some of them are reportedly the important transcription activators or suppressors of plant in responses to drought or ABA signals, e.g., ERF, WRKY, bHLH, and bZIP [68–71]. They might involve in the regulation of WUE in apple.

## Conclusion

We detected three stable QTLs on LG8, LG15, and LG16 for δ^13^C in apple under drought stress over two years, and validated them by KASP assay. Twenty-eight candidate genes in these QTLs were identified. They are involved in signaling, photosynthesis, response to stresses, carbohydrate metabolism, protein metabolism and modification, hormone metabolism and transport, transport, respiration, transcriptional regulation, and development regulation. These genes, especially those for photoprotection and relevant signal transduction, are potential candidates connected with WUE regulation in drought-stressed apple. These stable genetic loci and series of genes provided here serve as a foundation for further studies on MAS of high WUE and regulatory mechanism of WUE in apple exposed to drought conditions, respectively.

## Additional files

Additional file 1: **Table S1.** Primers used for qRT-PCR and KASP. (XLSX 244 kb)

Additional file 2: **Figure S1.** Linkage groups of parental and integrated maps for ‘Honeycrisp’ × ‘Qinguan’. The common markers between each parental map and integrated map were indicated by the green lines. (PPTX 271 kb)

Additional file 3: **Table S2.** List of genetic and physical map location of 10,172 SNP markers and their genotyping data among 350 F1 seedlings. (XLSX 169 kb)

Additional file 4: **Table S3.** Genes in the overlapping regions of three significant QTLs for δ^13^C across two years. (XLSX 165 kb)

Additional file 5: **Table S4.** Genes identified with GO annotations. (XLSX 166 kb)

Additional file 6: **Table S5.** Genes prioritised according to their GO functional annotations. (XLSX 166 kb)

Additional file 7: **Table S6.** Information *Arabidopsis* orthologs of candidate genes in apple under drought stress. **S6–1:** Summary information for homologous proteins in *Arabidopsis* of candidate genes; **S6–2:** Expression data for orthologs of candidate genes in roots of *Arabidopsis* under drought stress; **S6–3:** Expression data for orthologs of candidate genes in shoots of *Arabidopsis* under drought stress. (XLSX 166 kb)

Additional file 8: **Table S7.** Summary information for homologous proteins of candidate genes in STRING database. (XLSX 166 kb)

Additional file 9: **Table S8.** ID and descriptions of 45 genes tested by qRT-PCR and their relative expression levels in Qingguan and Honeycrisp under long-term drought stress condition. (XLSX 166 kb)

## Abbreviations

ABA: Abscisic acid
Chr: Chromosome
cM: centi-Morgen
DS: Drought-stressed
HC: Honeycrisp
IM: Interval mapping
KASP: Kompetitive allele specific PCR
LG: Linkage group
MAS: Marker assisted selection
MQM: Multiple QTL model
QG: Qinguan
qRT-PCR: Quantitative real-time PCR
QTLs: Quantitative trait loci
RADseq: Restriction site-associated DNA sequencing
ROS: Reactive oxygen species
SNP: Single nucleotide polymorphism
VPDB: Vienna peedee belemnite
WUE: Water use efficiency
WW: Well-watered
δ^13^C: Carbon isotope composition
