# Supplementary figures and images for: Mapping QTLs for water-use efficiency reveals the potential candidate genes involved in regulating the trait in apple under drought stress
Source: BMC Plant Biol. 2018 Jun 26;18:136. doi: 10.1186/s12870-018-1308-3 (PMC6019725; doi:10.1186/s12870-018-1308-3)

## Slide 1
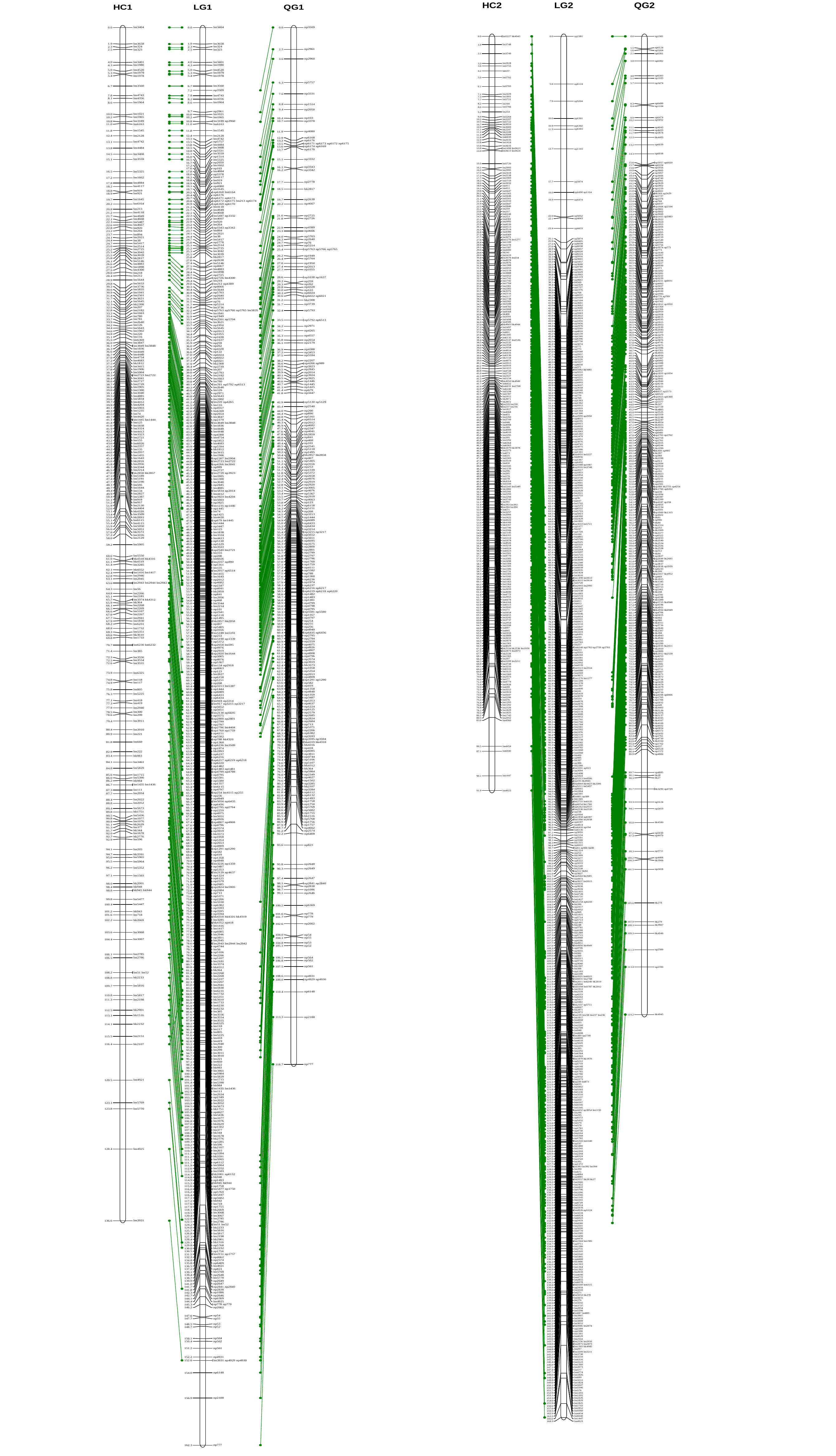

## Slide 2
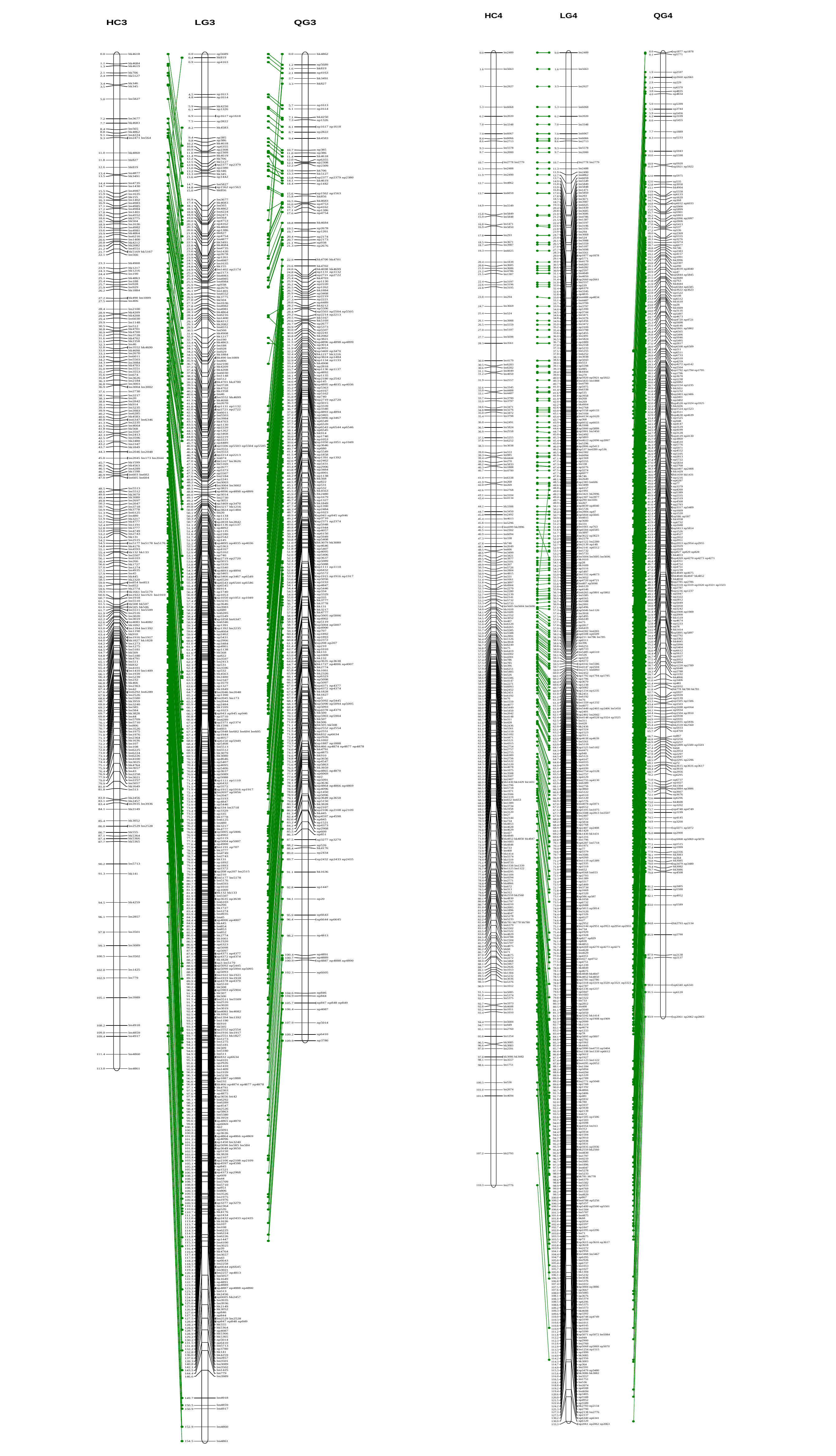

## Slide 3
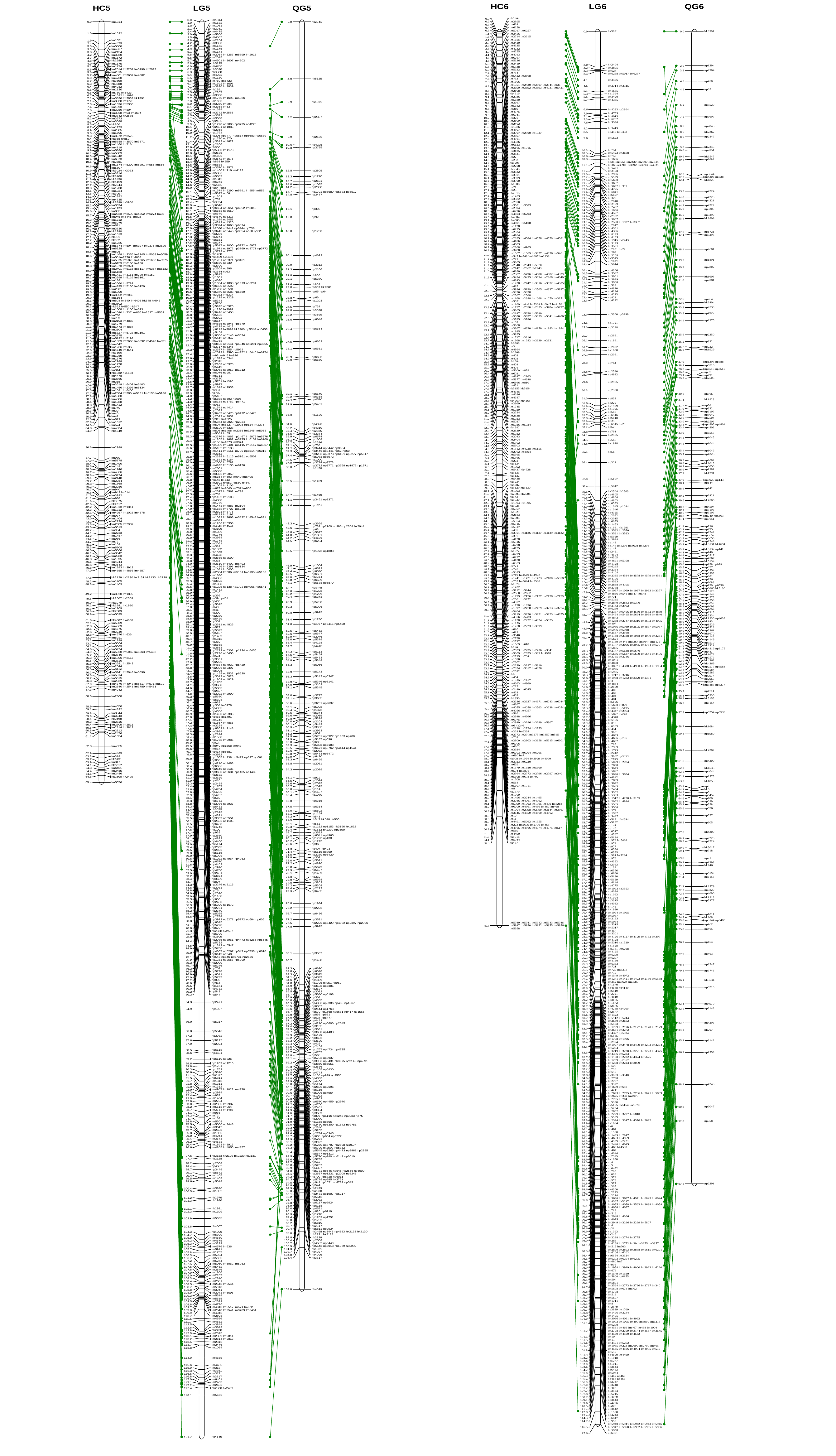

## Slide 4
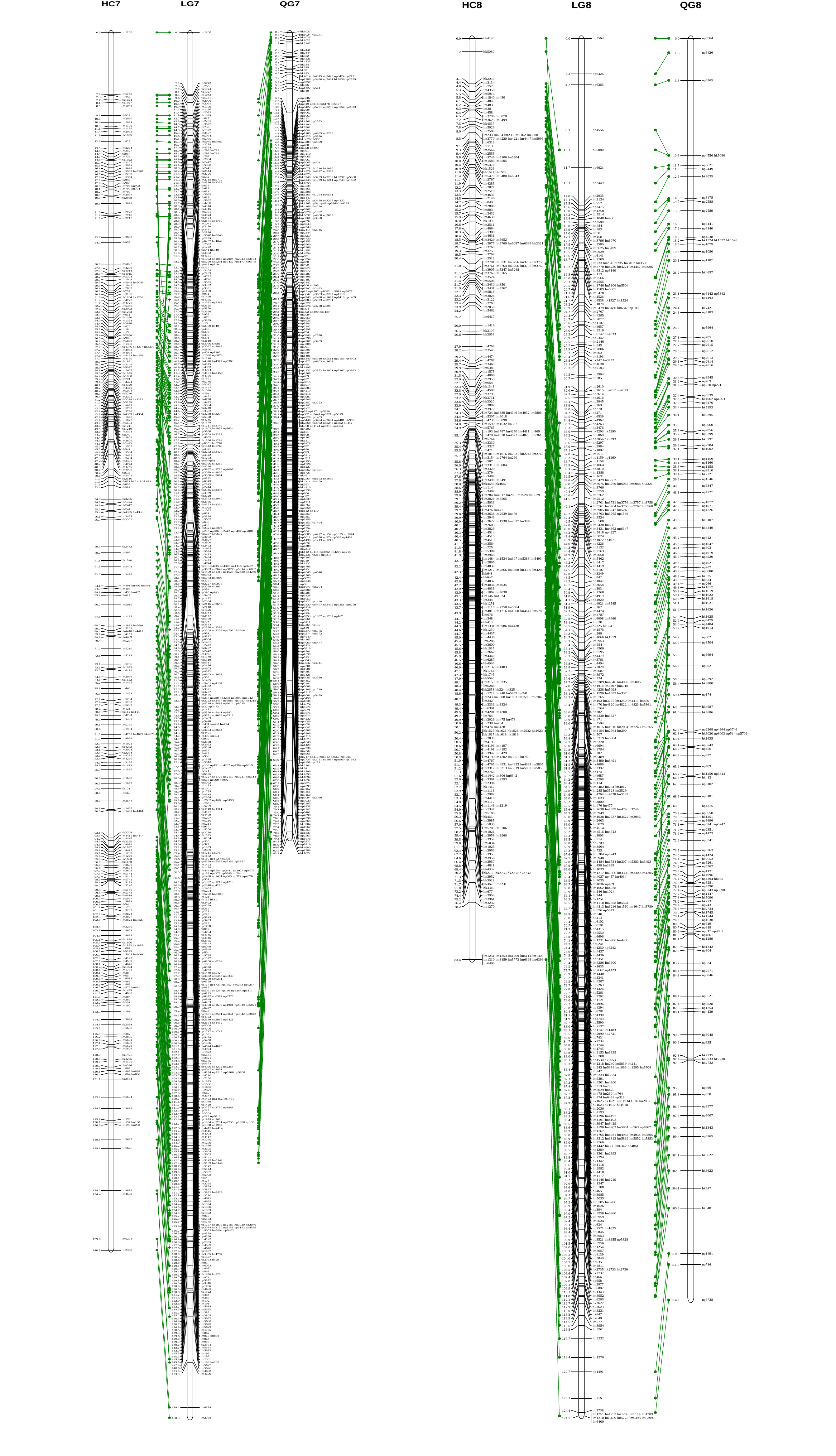

## Slide 5
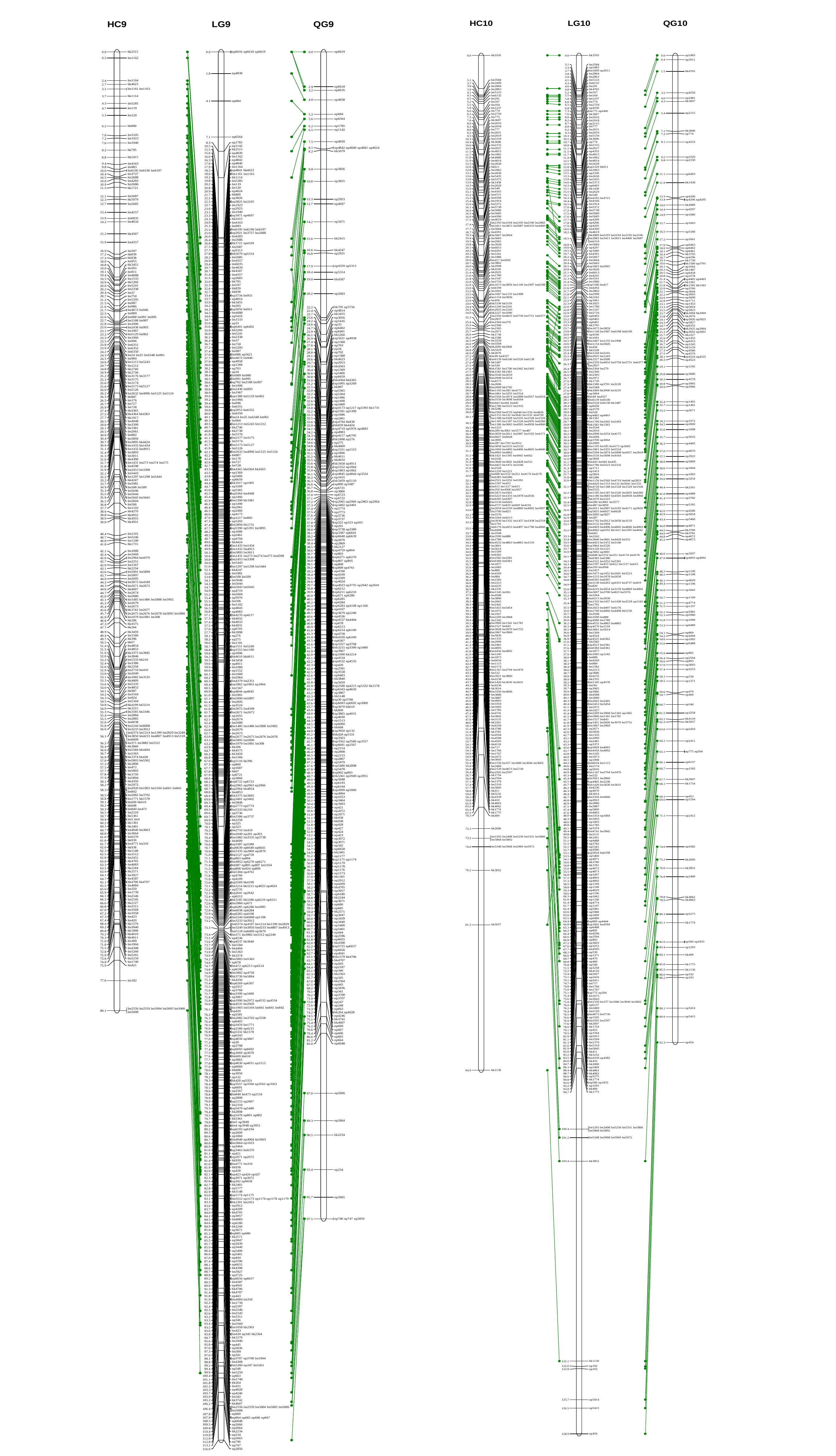

## Slide 6
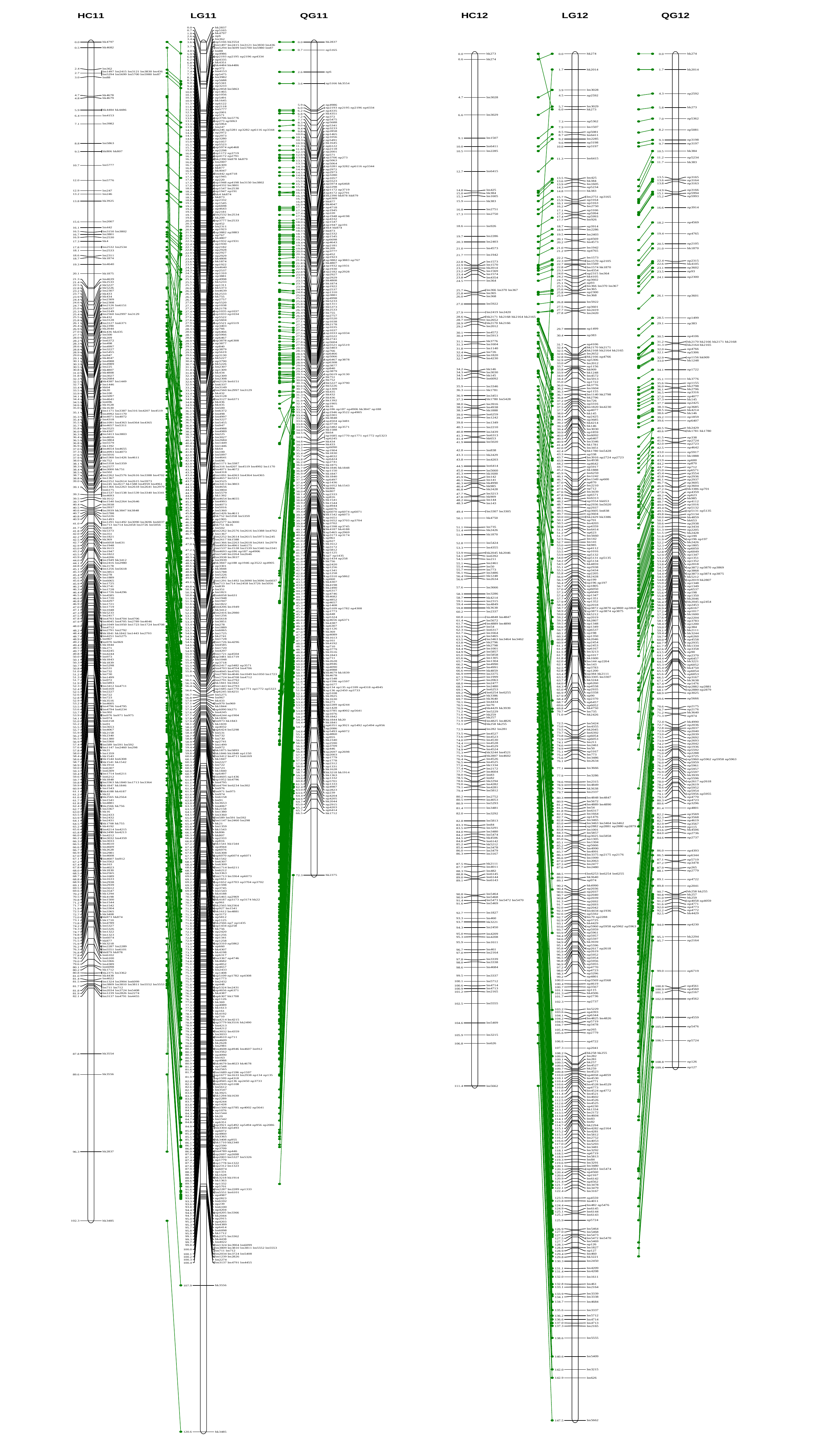

## Slide 7
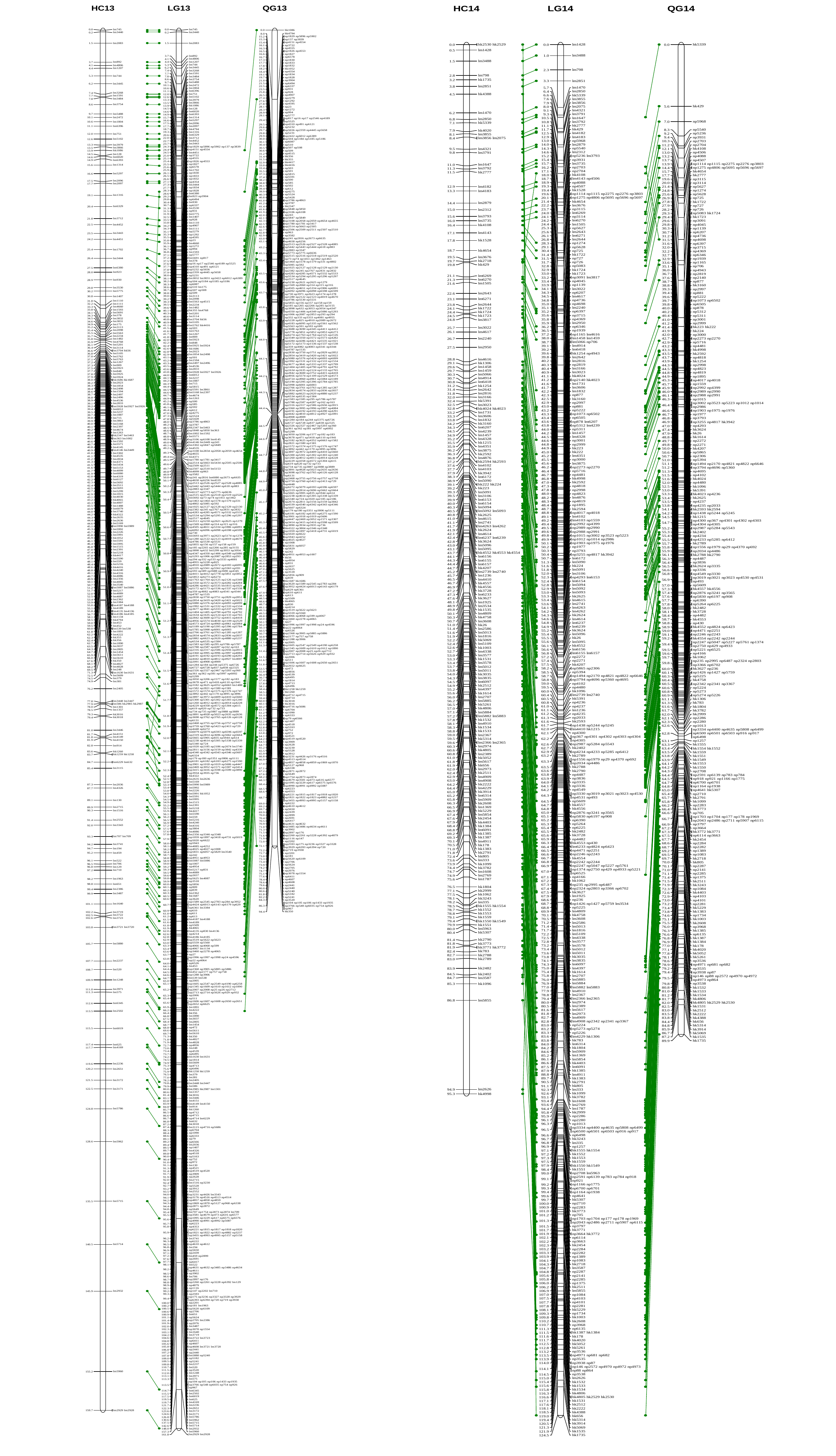

## Slide 8
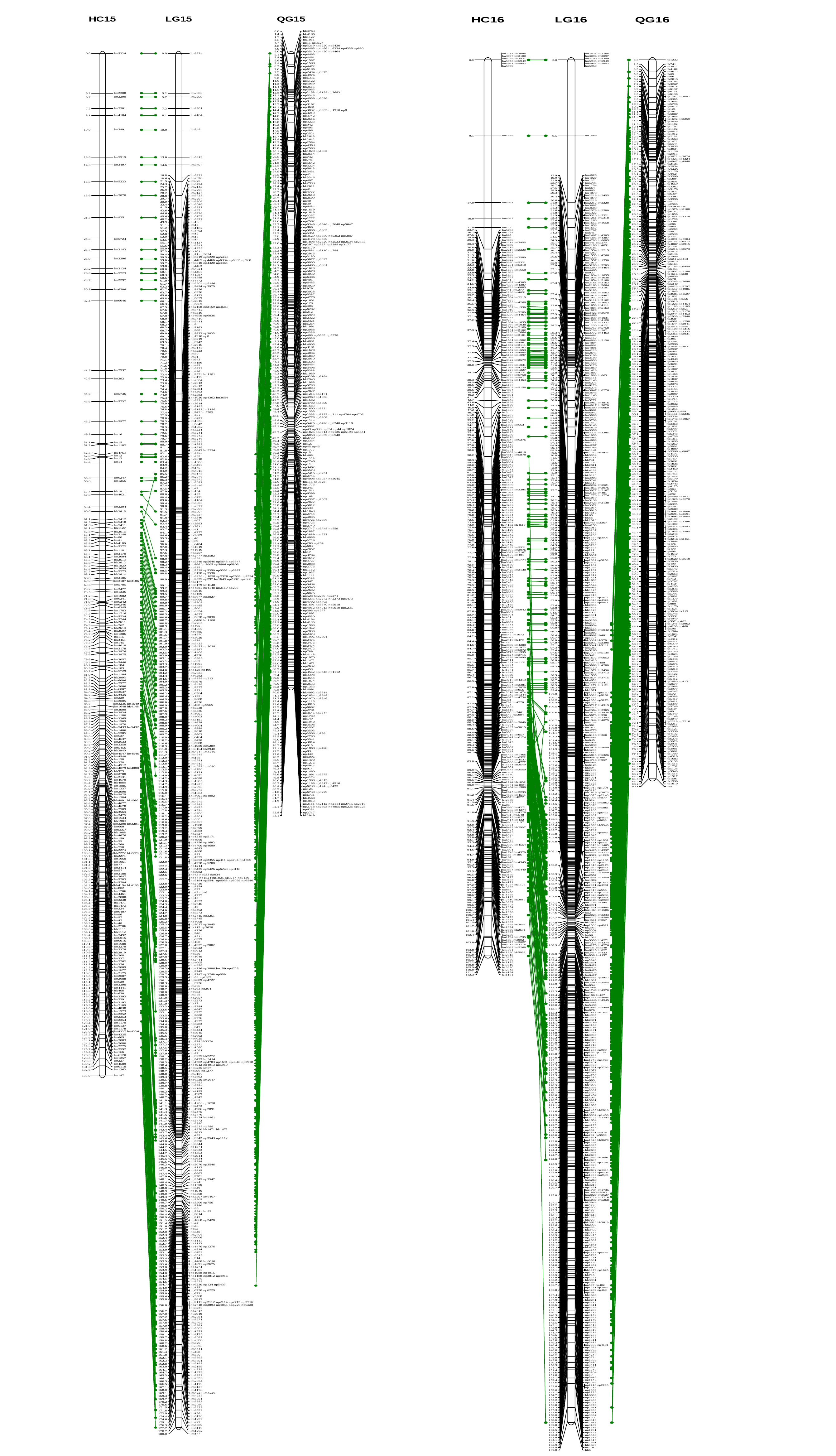

## Slide 9
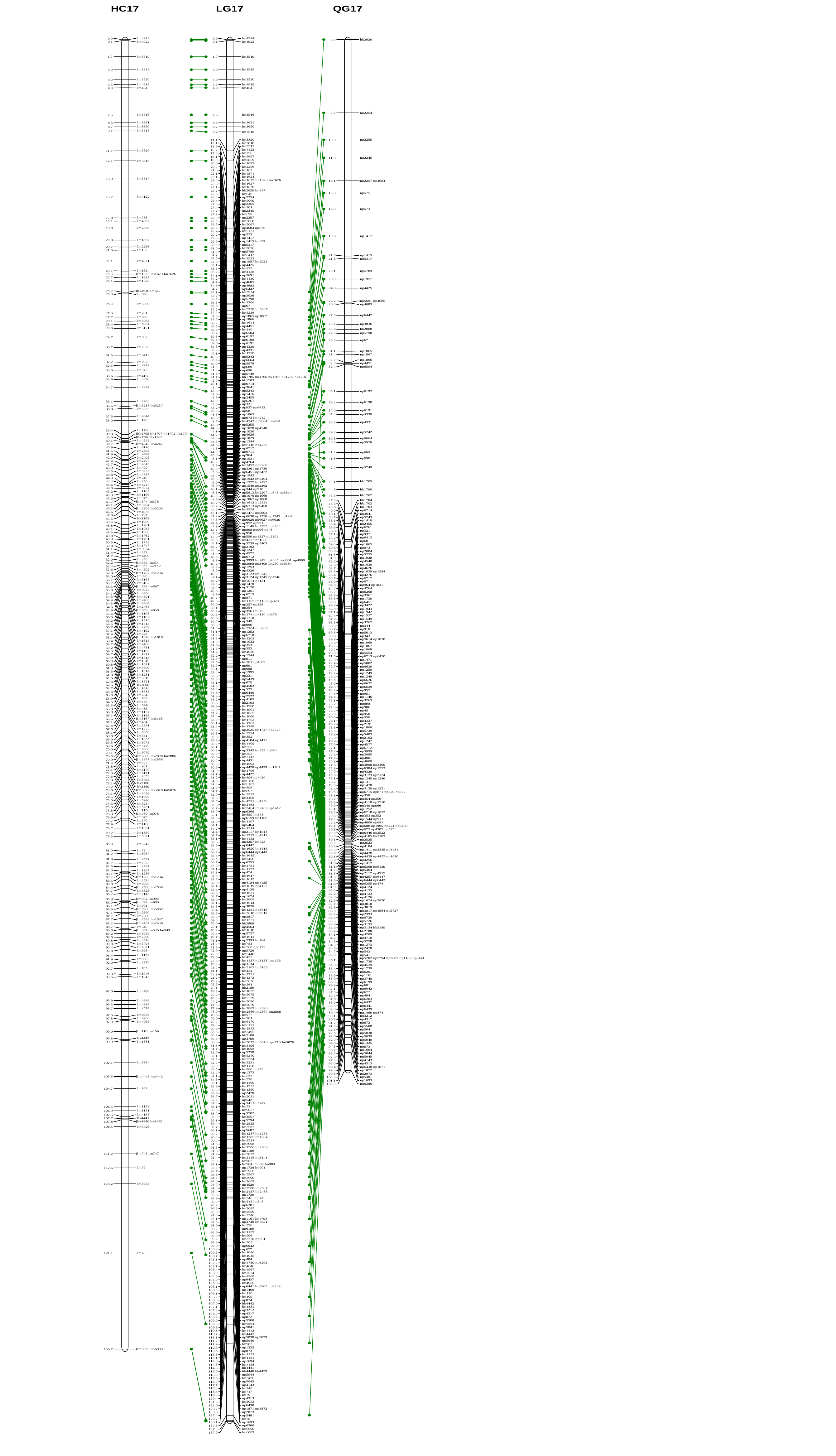

## Slide 10
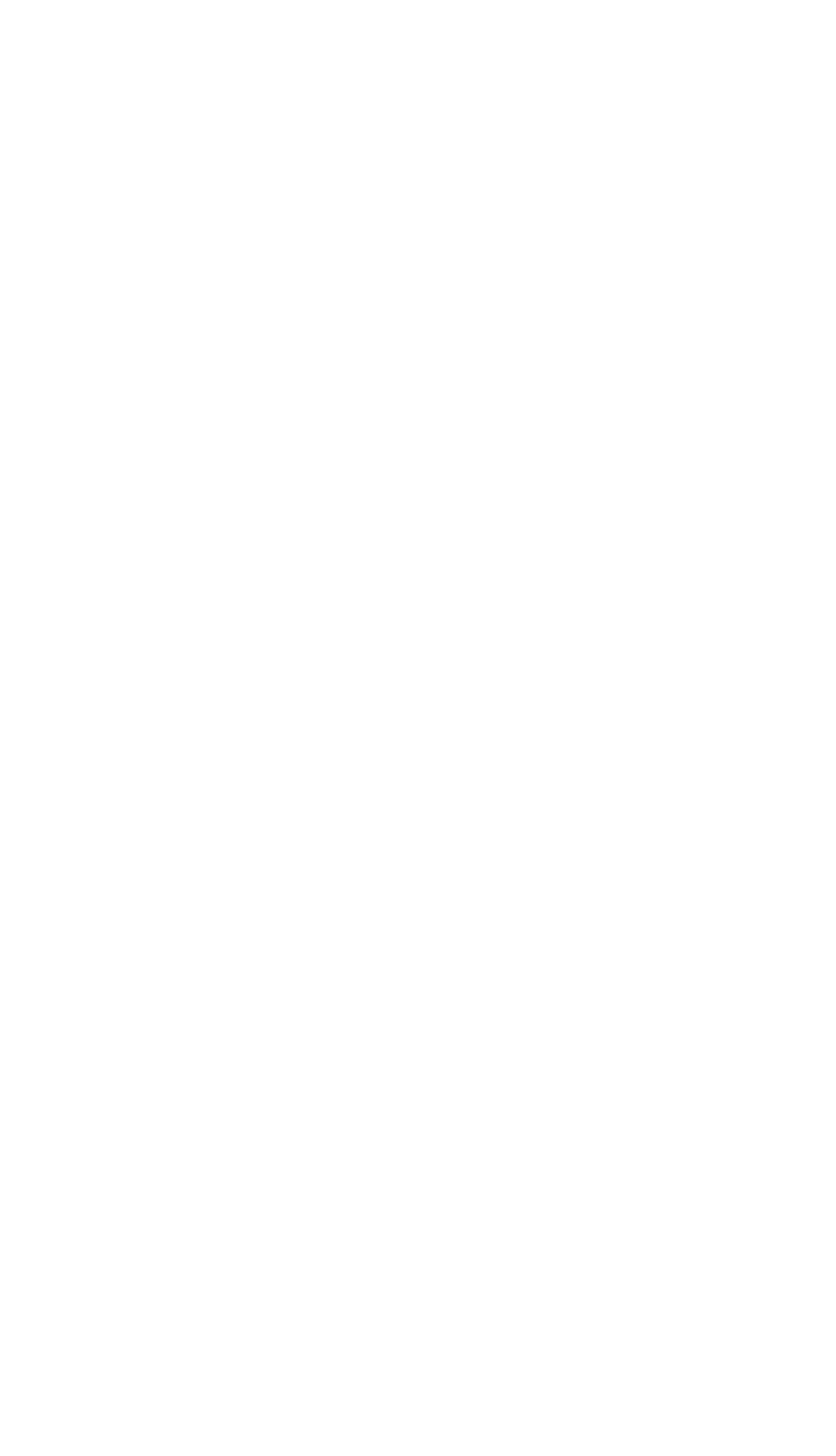

#

Supplement: Supplementary file 2 — Figure S1. Linkage groups of parental and integrated maps for ‘Honeycrisp’ × ‘Qinguan’. The common markers between each parental map and integrated map were indicated by the green lines. (PPTX 271 kb) [file 12870_2018_1308_MOESM2_ESM.pptx]
